# Forsythoside E Alleviates Liver Injury by Targeting PKM2 Tetramerization to Promote Macrophage M2 Polarization

**DOI:** 10.1002/advs.202514514

**Published:** 2025-11-14

**Authors:** Bingxin Wu, Xuechun Yu, Yanling Li, Lin An, Rongrong Zhang, Rong Zhang, Fan Zhang, Zhongqiu Liu, Caiyan Wang

**Affiliations:** ^1^ State Key Laboratory of Traditional Chinese Medicine Syndrome International Institute for Translational Chinese Medicine Guangzhou University of Chinese Medicine Guangzhou Guangdong 510006 China; ^2^ State Key Laboratory of Quality Research in Chinese Medicine Macau University of Science and Technology Macau SAR China

**Keywords:** forsythoside E, liver injury, pyruvate kinase M2, sepsis

## Abstract

Sepsis‐induced liver injury is closely associated with poor prognosis  in septic patients. In this study, through integrated approaches including high‐throughput virtual screening, target fishing technology, and atomic force microscopy (AFM) analysis, Forsythiaside E (FE) is first identified as a novel allosteric activator of pyruvate kinase M2 (PKM2) that promotes tetramer formation. Mutant protein construction combined with dynamic light scattering (DLS) and fluorescence resonance energy transfer (FRET) reveals FE binds to PKM2 K311 to promote tetramer formation. Employing Seahorse XF metabolic analyzers, real‐time single‐cell multi‐modal analysis systems, and transcriptome sequencing, it is revealed that FE‐mediated PKM2 tetramerization induces metabolic reprogramming in macrophages while suppressing STAT3 phosphorylation and subsequent NLRP3 transcriptional activation. In vivo assessments indicate that FE exhibited no significant multi‐organ toxicity. FE alleviates sepsis‐induced liver injury by promoting macrophage polarization toward the M2 anti‐inflammatory phenotype. Further validation through macrophage‐specific overexpression of PKM2 WT/K311A in mice confirms FE's mechanism of action. Collectively, this study elucidates the molecular mechanism by which FE alleviates sepsis‐associated liver injury through targeted PKM2 tetramerization and proposes an innovative metabolic‐epigenetic coordinated regulation strategy for sepsis‐related liver injury treatment.

## Graphical abstract

Here, the study shows for the first time that Forsythoside E (FE) can promote PKM2 tetramerization by binding to its K311 site. Furthermore, it induces metabolic reprogramming of macrophages and inhibits NLRP3 expression. Finally, it can alleviate sepsis‐induced liver injury by promoting M2 anti‐inflammatory polarization. In vivo experiments confirm that FE has good safety, which provides a new strategy for the treatment of sepsis‐induced liver injury.

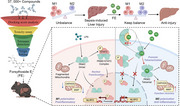

## 1 Introduction

Sepsis, a life‐threatening systemic inflammatory response syndrome triggered by infection, is pathologically characterized by immune hyperactivation and dysregulated cytokine storm, culminating in multiple organ dysfunction syndrome, septic shock, and ultimately mortality.^[^ ^1^ ^]^ Despite incremental progress in diagnostic modalities and therapeutic interventions in recent years, its persistently high mortality rate continues to pose a formidable challenge to global healthcare systems. The liver, functioning as a pivotal organ during septic progression, performs dual physiological imperatives of metabolic homeostasis and xenobiotic detoxification while serving as an immunological nexus.^[^ ^2^ ^]^ Paradoxically, this vital organ also ranks among the most vulnerable targets in sepsis pathophysiology. Sepsis‐associated liver injury not only critically compromises hepatic detoxification, metabolic regulation, and biosynthetic capacities but also perpetuates systemic inflammatory cascades, thereby exponentially elevating patient mortality risks.^[^ ^3^ ^]^

The pathological mechanisms underlying sepsis‐associated liver injury are complex, involving multifaceted interactions at cellular and molecular levels. Among these, macrophage hyperactivation is recognized as a central driver of hepatic injury. During sepsis, Kupffer cells in the liver become activated, releasing excessive pro‐inflammatory cytokines such as interleukins (ILs), tumor necrosis factor (TNF), and interferons (IFNs), which subsequently trigger hepatocyte secretion of acute‐phase proteins (APPs).^[^ ^4^ ^]^ These APPs further potentiate intrahepatic cytokine production, recruit immune cells, and exacerbate hepatocyte damage.^[^ ^5^ ^]^ Additionally, inflammatory monocytes are recruited to the liver during injury and differentiate into inducible nitric oxide synthase (iNOS)‐expressing pro‐inflammatory macrophages, which release large quantities of inflammatory mediators (e.g., TNF‐α, IL‐1β, IL‐6), intensifying local inflammation and promoting hepatocyte apoptosis and necrosis.^[^ ^6^, ^7^ ^]^ These inflammatory factors and APPs subsequently enter systemic circulation, inducing global immune dysregulation and establishing a vicious pathological cycle.^[^ ^4^ ^]^

Metabolic reprogramming of macrophages in inflammatory microenvironments constitutes a pivotal mechanism underlying septic liver injury. Under inflammatory stimuli, macrophages shift their energy metabolism from mitochondrial oxidative phosphorylation to glycolysis‐dependent pathways, a phenomenon termed the Warburg effect (aerobic glycolysis).^[^ ^8^ ^]^ This metabolic adaptation not only provides rapid energy supply for macrophages but also activates multiple inflammation‐related signaling pathways through the accumulation of glycolytic intermediates, thereby amplifying pro‐inflammatory cytokine release.^[^ ^9^, ^10^ ^]^ PKM2, serving as the glycolytic rate‐limiting enzyme, displays opposing functional regulation of macrophage metabolism and inflammatory responses through its dynamic oligomeric transition between dimeric and tetrameric states.^[^ ^8^, ^11^, ^12^, ^13^ ^]^ The dimeric PKM2 facilitates glycolytic intermediate accumulation, activates inflammatory signaling pathway (e.g., HIF‐1α, NF‐κB, and STAT3), and functions as both a transcriptional coactivator and protein kinase to promote inflammatory factors secretion.^[^ ^14^, ^15^ ^]^ Conversely, tetrameric PKM2 suppresses glycolysis while enhancing mitochondrial oxidative phosphorylation, thereby driving macrophage polarization from the pro‐inflammatory M1 phenotype toward the anti‐inflammatory M2 phenotype.^[^ ^16^ ^]^

Current therapeutic strategies for sepsis‐associated liver injury predominantly rely on antibiotic therapy and supportive care yet demonstrate limited efficacy in fundamentally reversing inflammatory response and metabolic dysregulation. Natural products play a significant role in treating inflammation through multiple mechanisms, particularly demonstrating remarkable potential in regulating inflammatory pathways, inhibiting oxidative stress, and balancing immune responses.^[^ ^17^ ^]^ For instance, Demethyleneberberin can alleviate liver inflammation by suppressing the NF‐κB and MAPK signaling pathways, and also exert anti‐fibrotic effects by inhibiting the activation of hepatic stellate cells.^[^ ^18^, ^19^ ^]^ Therefore, exploring natural drugs with multiple mechanisms of action has become a new research direction. *Forsythia suspensa* (Thunb.) Vahl, classical traditional Chinese medicine (TCM), exhibits broad‐spectrum anti‐inflammatory and immunomodulatory properties,^[^ ^20^, ^21^, ^22^, ^23^ ^]^ with its phenylethanoid glycosides (e.g., Forsythosides A, B) exhibiting promising hepatoprotective efficacy.^[^ ^20^, ^24^ ^]^ However, current pharmacological studies mainly focus on Forsythoside A and B, while the anti‐inflammatory mechanism of Forsythoside E (FE) is not fully understood.

In this study, we employed bioinformatic approaches and sepsis mouse models to demonstrate that PKM2 predominantly exists in a dimeric form with significantly elevated expression levels in the livers of sepsis mice. Through high‐throughput virtual screening of over 30 000 small molecular compounds, we identified FE and subsequently confirmed via molecular interaction assays that this compound directly targets PKM2 to promote its tetrameric assembly. Mechanistically, FE‐mediated PKM2 tetramerization induces metabolic reprogramming in macrophages while simultaneously suppressing STAT3 phosphorylation, thereby reducing NLRP3 transcriptional activation. This cascade ultimately drives macrophage polarization toward the M2 phenotype, ameliorating sepsis‐induced liver injury. Our findings not only identify FE as a novel allosteric modulator of PKM2, offering a promising therapeutic strategy for sepsis‐associated liver damage, but also provide a methodological framework for investigating the molecular targets and mechanisms of bioactive components in traditional Chinese medicine.

## 2 Results

### 2.1 PKM2 Plays a Key Role in the Process of Sepsis‐Induced Liver Injury

**Figure 1:**
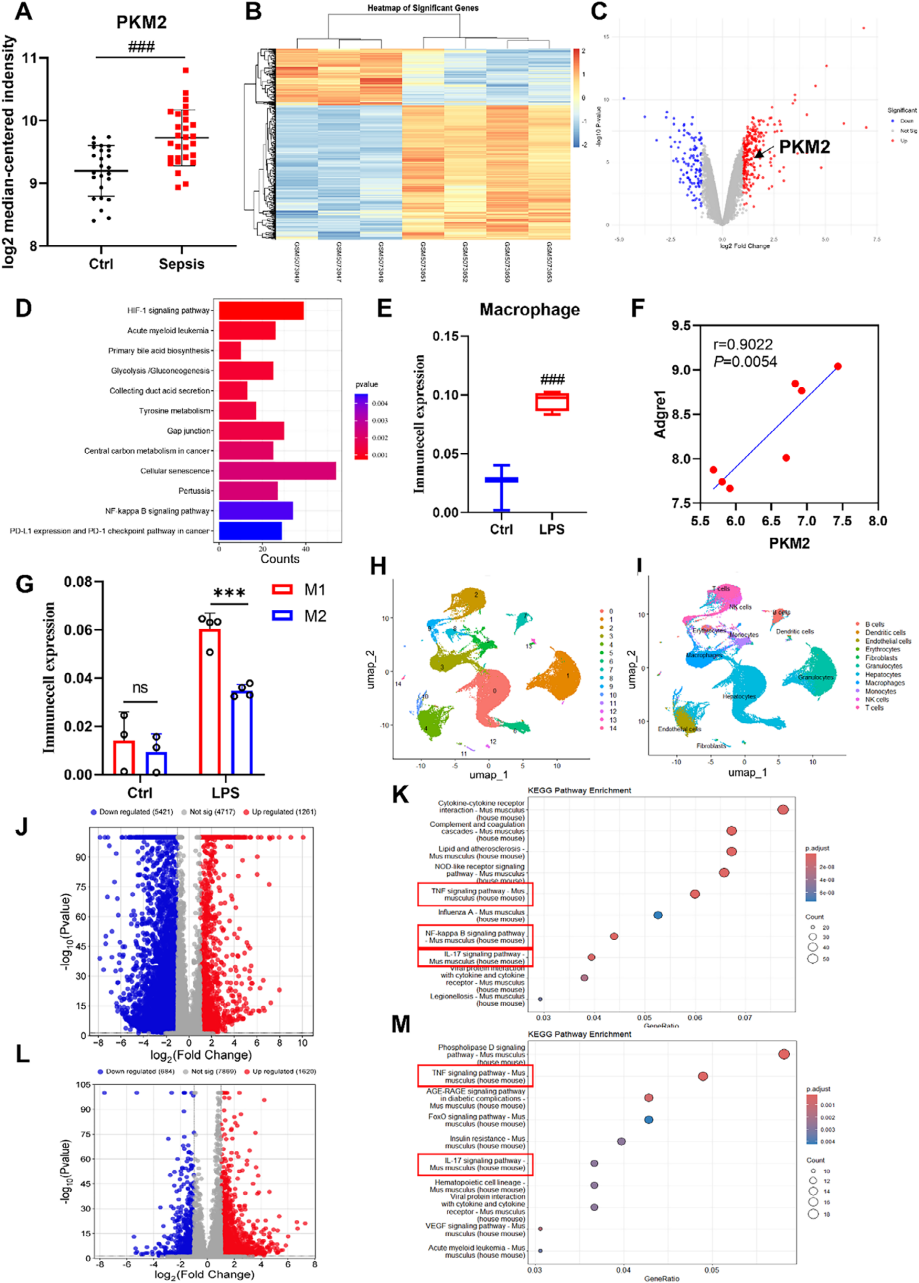
PKM2 Aggravates Sepsis‐Induced Liver Injury. A) Analysis of PKM2 gene expression in normal individuals and sepsis patients using GEO 2R on dataset GSE57065. B) Heatmap of differentially expressed genes in the GSE166488 dataset. C) Volcano plot of differentially expressed genes in the GSE166488 dataset. D) KEGG pathway enrichment analysis of differentially expressed genes in the GSE166488 dataset. E) Macrophage infiltration in liver tissues of Ctrl and LPS‐treated mice. F) Correlation between PKM2 mRNA expression and Adgre1 mRNA expression in mouse liver tissues. G) Infiltration of M1 and M2 macrophages in liver tissue of Ctrl and LPS‐treated mice. H,I) UMAP analysis of the GSE254497 dataset. J) Volcano plot of differentially expressed genes in macrophages between septic mice and normal mice, with K) KEGG analysis of upregulated genes. L) Volcano plot of differentially expressed genes in macrophages between myeloid specific *Pkm* knockout sepsis mice and non‐knockout sepsis mice, with M) KEGG analysis of downregulated genes. Data are expressed as the mean ± SD. ^###^ *p*<0.001 versus the control group; ^***^ *p* < 0.001 versus M2 group.

To determine whether PKM2 plays a significant role in sepsis, we first analyzed the GSE57065 dataset from the GEO database. The results revealed that the gene expression level of *PKM2* was significantly elevated in sepsis patients compared to healthy individuals (**Figure** **1**A), suggesting that PKM2 may play a critical role in the pathogenesis of sepsis. To further confirm the role of PKM2 in the liver during sepsis‐induced liver injury, we analyzed the GSE166488 dataset and found that PKM2 was highly expressed in the livers of septic mice compared to normal mice (Figure 1B,C). Additionally, KEGG pathway enrichment analysis of differentially expressed genes identified Glycolysis/Gluconeogenesis and HIF‐1α signaling pathways (Figure 1D), in which PKM2 serves as a key regulator. These findings collectively indicate that PKM2 is involved in the pathogenesis of sepsis‐induced liver injury. Using the ImmuCell AI algorithm, we observed a significant increase in macrophage infiltration in the livers of septic mice (Figure 1E), which exhibited a clear correlation with elevated PKM2 expression (Figure 1F). Further analysis of macrophage polarization phenotypes revealed that, compared to normal mice, the numbers of both M1 and M2 macrophages were increased in septic mice, but the number of M1 macrophages was significantly higher than that of M2 macrophages (Figure 1G). To further investigate the relationship between PKM2 and macrophages, we analyzed the GSE254497 dataset and extracted macrophage‐related data (Figure 1H,I). By comparing with normal mice, differential genes of macrophages in the liver of septic mice were obtained, and KEGG enrichment analysis was performed on the upregulated genes(Figure 1J). The results demonstrated that genes upregulated in macrophages within septic mouse liver tissues were primarily enriched in immune‐inflammatory signaling pathways, such as the TNF signaling pathway, NF‐κB signaling pathway, and IL‐17 signaling pathway (Figure 1K). Next, we will compare the differentially expressed genes between myeloid specific *Pkm* knockout sepsis mice and non‐knockout sepsis mice and perform KEGG enrichment analysis on the down regulated genes (Figure 1L). In contrast, genes downregulated in macrophages from myeloid‐specific *Pkm* knockout mice were predominantly enriched in the TNF and NF‐κB signaling pathways (Figure 1M). These findings suggest that PKM2 is critically involved in activating macrophage‐associated immune‐inflammatory signaling pathways during liver injury. Collectively, these results suggest that PKM2 may be a key therapeutic target in sepsis‐induced liver injury.

### 2.2 Virtual Screening was used to Obtain Potential Compounds for Promoting PKM2 Tetramerization

**Figure 2:**
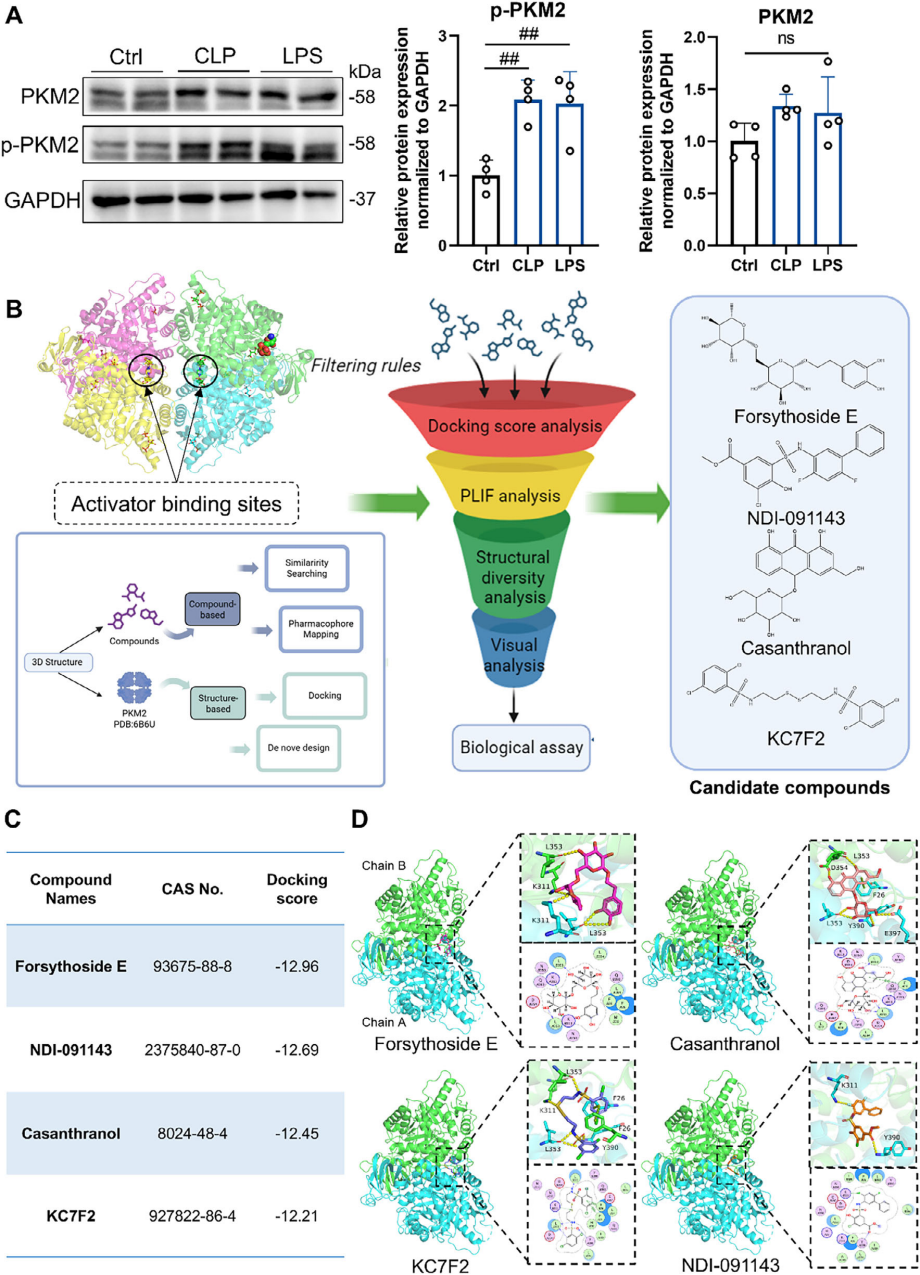
High Throughput Virtual Screening to Search for Allosteric Activators. A) Expression of PKM2 and p‐PKM2 proteins in liver tissues of Ctrl, CLP, and LPS‐treated mice. B) Schematic diagram of virtual screening process targeting the agonist‐binding site of PKM2. C) Names, CAS numbers, and docking scores of the top four candidate compounds. D) Molecular docking conformations of the four compounds with PKM2. Data are expressed as the mean ± SD. ^##^ *p* < 0.01 versus the control group.

Previous studies have demonstrated that PKM2 exists in dimeric and tetrameric forms, which exert distinct functional roles. To further investigate the aggregation states of PKM2 during sepsis, we established two murine models of sepsis‐induced liver injury via intraperitoneal LPS injection and CLP. Western blot analysis revealed that both models exhibited significantly increased PKM2 expression compared to normal mice, accompanied by elevated levels of phosphorylated PKM2 (p‐PKM2) (**Figure** **2**A). Phosphorylated PKM2 predominantly exists in the dimeric form, which is known to exhibit protein kinase activity. These findings suggest that PKM2 likely functions primarily in its dimeric form during sepsis. To identify potent compounds capable of inhibiting the dimeric form of PKM2 and promoting its tetramerization, a structure‐based virtual screening strategy targeting the agonist binding site of PKM2 was employed to screen over 30,000 small molecule compounds (Figure 2B). Based on docking scores, the top four compounds were ultimately selected (Figure 2C). Visual inspection of the molecular binding conformations with the protein revealed that all four compounds could bind to the agonist‐binding site of PKM2. The A and B chains of the protein are blue and green Cartoon structures, respectively. The yellow dotted line indicates hydrogen bonds (Figure 2D).

### 2.3 FE Promotes PKM2 Tetramerization and Inhibits Macrophage Inflammation

**Figure 3:**
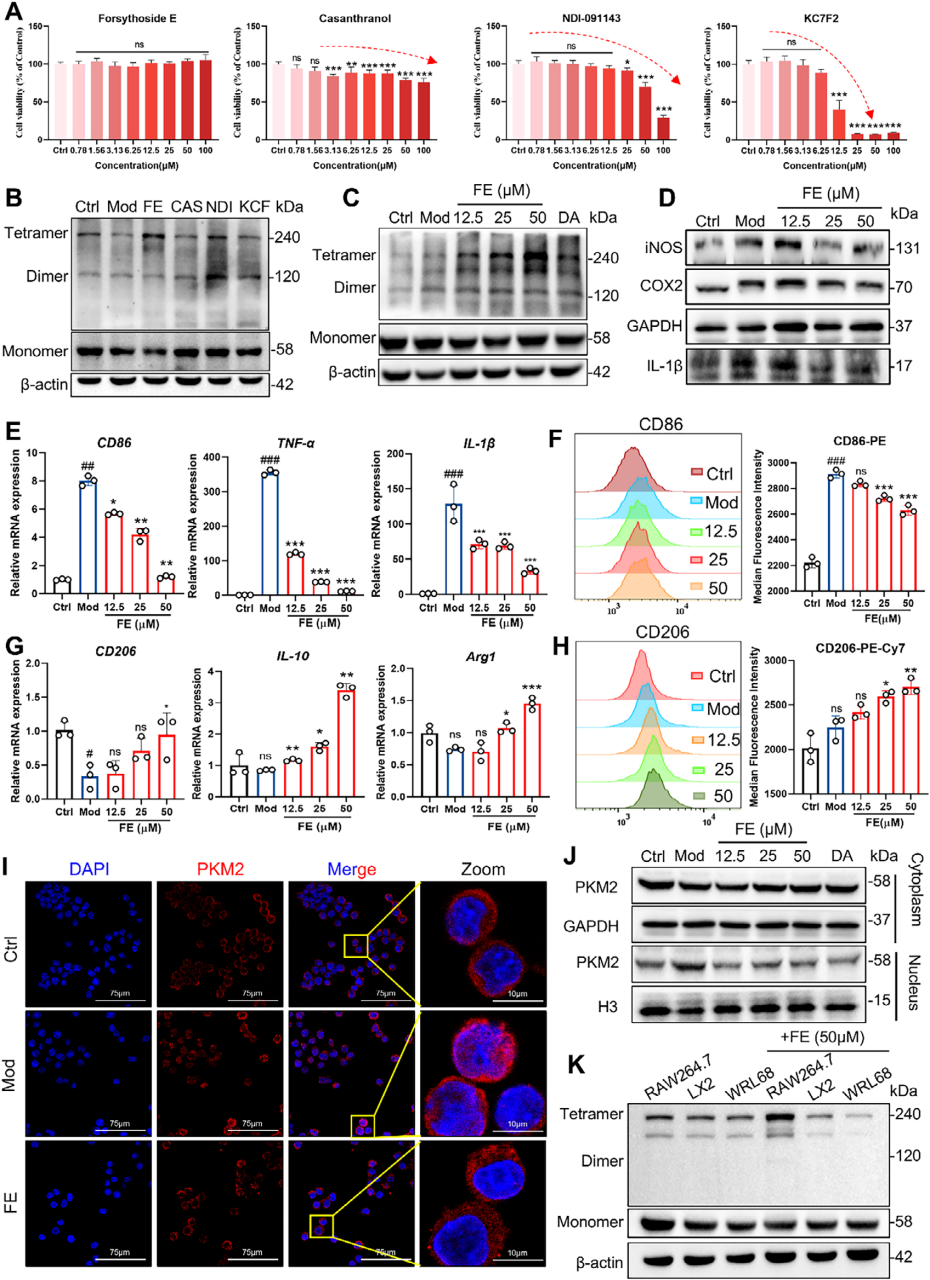
FE is a Potential Allosteric Activator. A) Cytotoxicity of four compounds on RAW264.7 cells assessed by MTT assay. B) DSS crosslinking analysis of PKM2 multimers in RAW264.7 cells treated with four compounds. C) Expression of PKM2 multimers in LPS‐induced macrophages treated with FE (12.5, 25, 50 µm) or DA (DASA‐58, 20 µm). D) Western blot analysis of inflammation‐related proteins (iNOS, COX2, IL‐1β) in LPS‐induced RAW264.7 cells. E) mRNA expression of M1 (CD86, TNF‐α, IL‐1β) and G) M2 (CD206, IL‐10, ARG1) polarization markers in LPS‐induced RAW264.7 cells treated with FE. F) The expression of CD86 and H) CD206 on the cell surface was detected by flow cytometry. I) Immunofluorescence localization of PKM2 in cytoplasm and nucleus of FE‐treated RAW264.7 cells. J) Western blot analysis of PKM2 expression in cytoplasmic and nuclear fractions of RAW264.7 cells. K) PKM2 tetramer protein expression levels in different cells. Data are expressed as the mean ± SD. ^#^ *p* < 0.05, ^##^ *p* < 0.01, ^###^ *p* < 0.001 versus the control group; ^*^ *p* < 0.05, ^**^ *p* < 0.01, ^***^ *p* < 0.001 versus the model group.

To further identify the most effective compound of the four candidates, we first assessed their cytotoxicity. The results showed that FE exhibited the lowest cytotoxicity (**Figure** **3**A). Subsequently, by examining the multimeric forms of PKM2, we found that, compared to the other three compounds, FE effectively promoted the formation of PKM2 tetramers in RAW264.7 cells (Figure 3B). Based on these findings, we selected FE as the focus of our subsequent studies. Further research revealed that FE dose‐dependently enhanced the expression of the tetrameric form of PKM2 in macrophages (Figure 3C). Western blot analysis also demonstrated that FE effectively reduced the expression levels of inflammation‐related proteins in macrophages (Figure 3D). The qPCR results showed that the expression of M1 polarization‐related genes, such as *Cd86*, *Tnf‐α*, and *IL‐1β*, which were increased in macrophages by LPS induction, was significantly reduced after treatment with FE (Figure 3E). The expression of CD86 on the cell surface was further detected by flow cytometry, and the expression of CD86 on the cell surface was significantly increased by LPS, while FE could reduce its expression in a dose‐dependent manner (Figure 3F). In contrast, M2 polarization‐related genes, including *Cd206*, *IL‐10*, and *Arg1*, were upregulated (Figure 3G). There was also an increase in CD206 expression as measured by flow cytometry (Figure 3H). The dimeric form of PKM2 can translocate to the nucleus, promoting the expression of inflammation‐related genes. Immunofluorescence and nuclear‐cytoplasmic fractionation experiments revealed that FE significantly reduced the nuclear distribution of PKM2 (Figure 3I,J). Meanwhile, this study compared the effect of FE on PKM2 tetramer formation in different cell lines. The results showed that FE effectively promoted PKM2 tetramerization in RAW264.7 cells, whereas no significant promotion was observed in LX2 and WRL68 cells (Figure 3K). These results indicate that FE effectively promotes PKM2 tetramerization and reduces the expression of inflammation‐related proteins in macrophages.

### 2.4 Dual‐Probe Target Fishing and Interaction Experiments Confirmed that FE Directly Targeted PKM2

**Figure 4:**
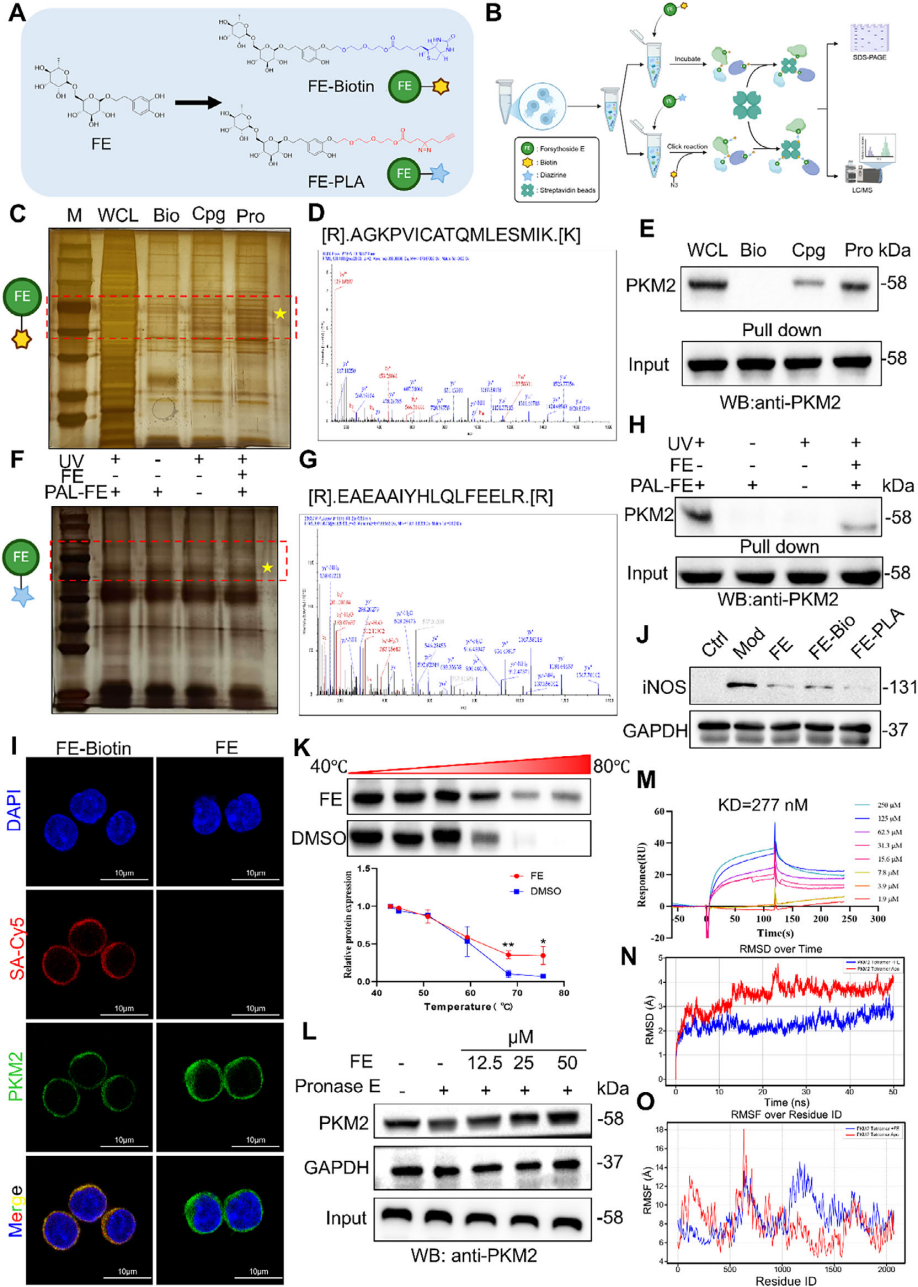
FE Directly Targets PKM2. A) Chemical structures of two FE probes. B) Schematic diagram of target fishing assay. C) Silver staining results of FE‐Biotin target fishing. D) Mass spectrometry identification of PKM2 protein from FE‐Biotin fishing. E) Western blot validation of PKM2 protein from FE‐Biotin fishing. F) Silver staining results of FE‐PLA target fishing. G) Mass spectrometry identification of PKM2 protein from FE‐PLA fishing. H) Western blot validation of PKM2 protein from FE‐PLA fishing. I) Immunofluorescence co‐localization of FE‐Biotin with PKM2 protein. J) Inhibitory effect of different probes on iNOS protein expression. K) CETSA analysis of thermal stability of PKM2 protein. L) DARTS analysis of PKM2 protein expression. M) SPR analysis of binding affinity between FE and PKM2 protein. N) RMSD and O) RMSF analyzed by molecular dynamics simulation to evaluate the stability of PKM2 Tetramer Apo (blue) and PKM2 Tetramer+FE (red).Data are expressed as the mean ± SD. ^#^ *p* < 0.05, ^##^ *p* < 0.01, ^###^ *p* < 0.001 versus the control group; ^*^ *p* < 0.05, ^**^ *p* < 0.01, ^***^ *p* < 0.001 versus the model group.

To further confirm whether FE directly targets PKM2, we chemically modified FE by adding a biotin tag and a photoaffinity tag (**Figure** **4**A) and performed target fishing assays (Figure 4B). Silver staining revealed a distinct band between 55 and 70 kDa (Figure 4C), and LC‐MS analysis identified peptide fragments associated with PKM2 (Figure 4D). Subsequent pull‐down demonstrated that biotin‐tagged FE could directly bind to the PKM2 protein (Figure 4E). Similarly, photoaffinity‐tagged FE was also able to pull down the PKM2 protein (Figure 4F–H). Immunofluorescence results showed that FE colocalized with PKM2 in the cytoplasm (Figure 4I). Compared with FE, both probes inhibited iNOS protein expression levels, indicating that the two tags did not affect the anti‐inflammatory effect of FE (Figure 4J). Additionally, CETSA and DARTS results demonstrated that FE significantly enhanced the stability of the PKM2 protein (Figure 4K,L). To further evaluate the binding affinity between FE and the PKM2 protein, we conducted SPR experiments. The results revealed a binding affinity of 277 nM between PKM2 and FE, indicating that FE could directly and effectively bind to PKM2 (Figure 4M). Molecular dynamics simulation results demonstrated that FE binding to the PKM2 tetramer reduced both RMSD and RMSF, indicating that FE enhanced the stability of the PKM2 tetramer (Figure 4N,O). These findings collectively confirm that FE directly targets and binds to PKM2.

### 2.5 PKM2 K311 is the Key Amino Acid that Mediates the Binding of FE

**Figure 5:**
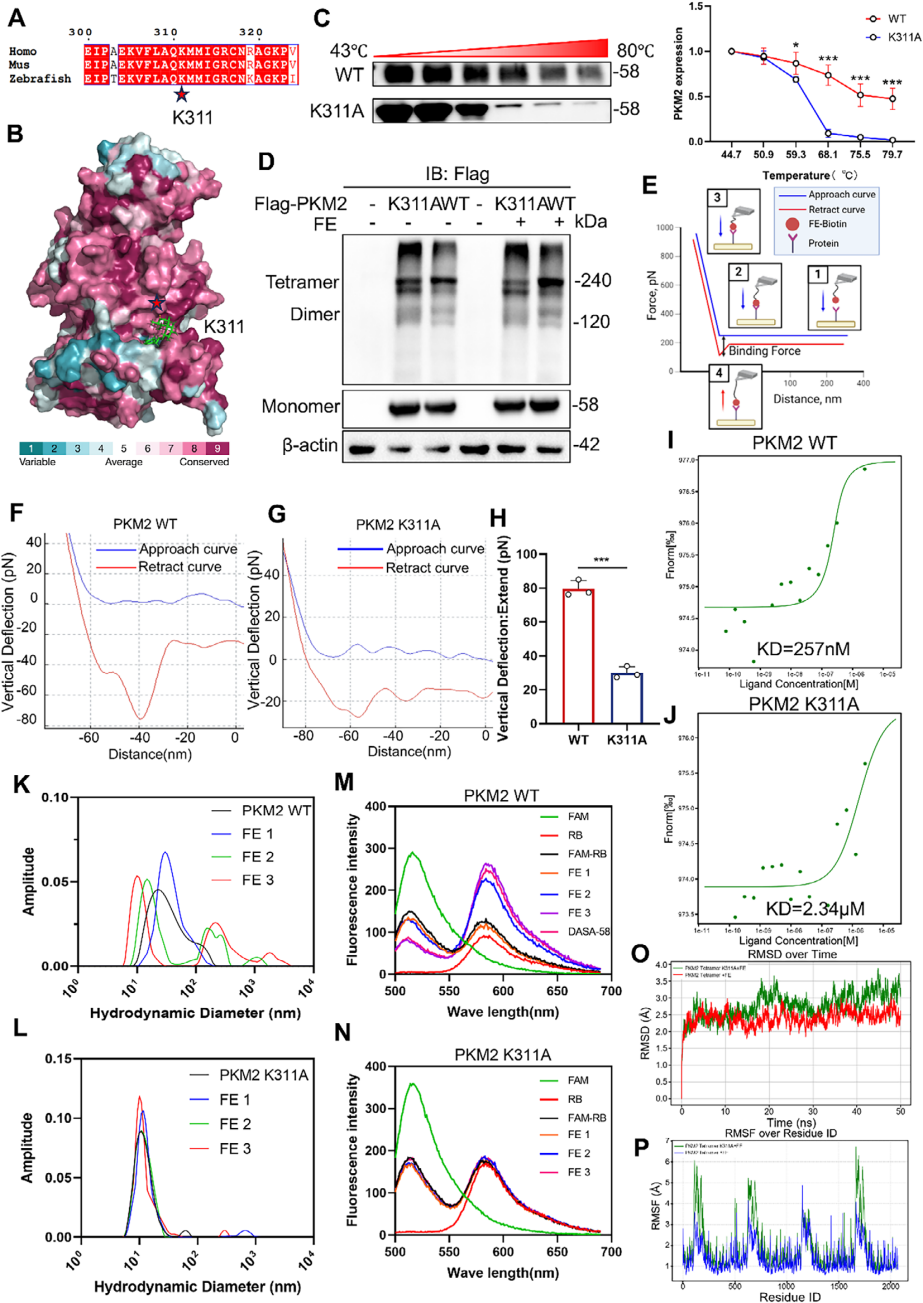
FE Binds Directly to K311 of PKM2. A) Sequence conservation analysis of PKM2 protein using ESPript 3.0. B) Consurf analysis of PKM2 protein sequence conservation. C) CETSA analysis of interactions between FE and PKM2 recombinant proteins. D) DSS crosslinking analysis of PKM2 multimers in 293T cells. E) Schematic diagram of atomic force microscopy force spectroscopy analysis. F–H) Force spectroscopy analysis of PKM2 WT and K311A recombinant proteins. I) MST analysis between FE and PKM2 WT. J) MST analysis between FE and PKM2 K311A. K) Dynamic light scattering analysis of particle size of PKM2 WT. L) Dynamic light scattering analysis of particle size of PKM2 K311A. M) FRET analysis of aggregation of PKM2 WT. N) FRET analysis of aggregation of PKM2 K311A. O) RMSD and P) RMSF analyzed by molecular dynamics simulation to evaluate the stability of PKM2 Tetramer K311A+FE (green) and PKM2 Tetramer+FE (red/blue). Data are expressed as the mean ± SD. ^#^ *p* < 0.05, ^##^ *p* < 0.01, ^###^ *p* < 0.001 versus the control group; ^*^ *p* < 0.05, ^**^ *p* < 0.01, ^***^ *p* < 0.001 versus the model group.

To further confirm the binding site of FE on the PKM2 protein, based on the molecular docking results between FE and PKM2, we hypothesized that K311 of PKM2 might be a critical amino acid. Conservation analysis of PKM2 K311 revealed that this site is conserved across multiple species (**Figure** **5**A,B). After mutating this site to alanine and obtaining the recombinant protein, we performed CETSA experiments, which showed that, compared to the wild‐type (WT) protein, FE could not effectively enhance the thermal stability of the mutant protein (Figure 5C). We overexpressed PKM2 WT and PKM2 K311A proteins in cells and examined the multimeric forms of PKM2. The results showed that FE could not effectively increase the expression of PKM2 K311A tetramers (Figure 5D). To further assess whether this mutation weakened the binding between FE and PKM2, AFM revealed that the binding force between FE and PKM2 K311A was significantly reduced compared to the WT protein (Figure 5E–H). Meanwhile, the MST results showed that the KD value of FE binding to PKM2 K311A was significantly higher than that for PKM2 WT (Figure 5I,J). DLS results demonstrated that FE effectively increased the particle size of PKM2 WT (Figure 5K), and FRET experiments confirmed that FE promoted the aggregation of PKM2 WT (Figure 5M). However, these effects were not observed with PKM2 K311A (Figure 5L,N), suggesting that the mutation at this site reduced the ability of FE to promote PKM2 tetramerization. Molecular dynamics simulations showed that FE did not reduce the RMSD and RMSF of PKM2 K311A tetramer (Figure 5O,P). Collectively, these results demonstrate that PKM2 K311 mediates the binding of FE to PKM2.

### 2.6 FE Inhibits Macrophage Glycolysis and Restores Mitochondrial Function by Promoting PKM2 Tetramerization

**Figure 6:**
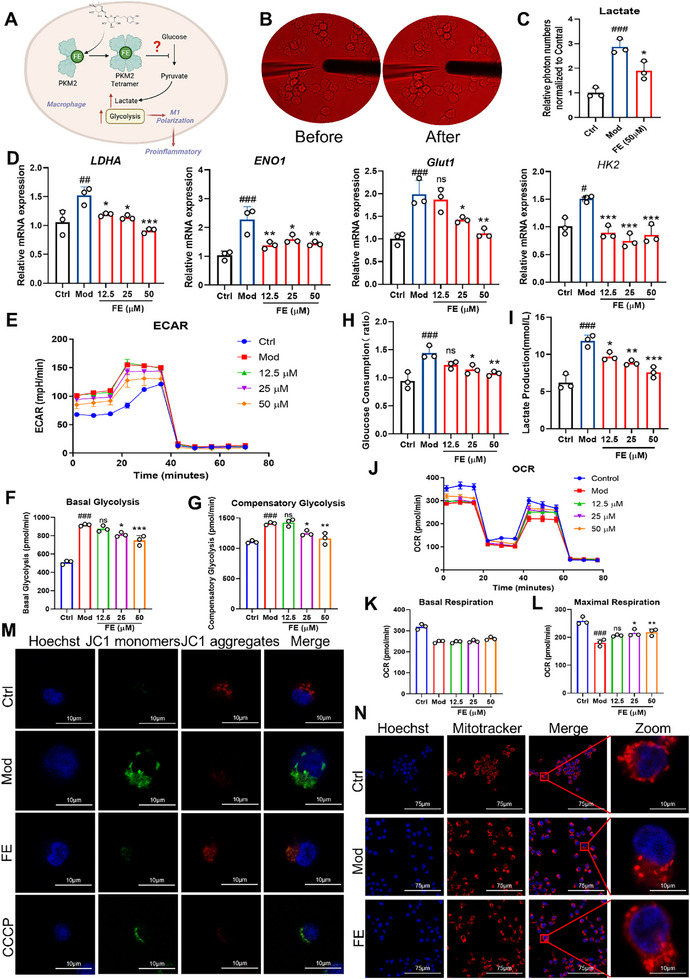
FE Promotes Metabolic Reprogramming in Macrophages. A) Hypothesis of FE inhibiting glycolysis in macrophages. B) Morphological changes of cells before and after single‐cell injection. C) Single‐cell analysis of lactate content in cells. D) mRNA expression of glycolysis‐related proteins. E–G) ECAR measurements, including basal and compensatory glycolysis levels. H) Glucose uptake in RAW264.7 cells. I) Lactate secretion in RAW264.7 cells. J–L) OCR measurements, including basal respiration and maximal respiration. M) JC1 staining analysis of mitochondrial membrane potential in RAW264.7 cells. N) Mitotracker analysis of mitochondrial morphology in RAW264.7 cells. Data are expressed as the mean ± SD. ^#^ *p* < 0.05, ^##^ *p* < 0.01, ^###^ *p* < 0.001 versus the control group; ^*^ *p*<0.05, ^**^ *p*<0.01, ^***^ *p*<0.001 versus the model group.

Upon activation, macrophages primarily rely on glycolysis for their metabolic needs. PKM2, a rate‐limiting enzyme in glycolysis, increases in its tetrameric form. This form of PKM2 inhibits glycolysis. We have previously established that FE targets and binds to PKM2, promoting its tetramerization. Therefore, we hypothesized that the anti‐inflammatory activity of FE is closely related to its inhibition of glycolysis in macrophages (**Figure** **6**A). First, we assessed lactate secretion in macrophages by directly injecting a lactate detection reagent and measuring photon counts. The results showed that FE effectively suppressed LPS‐induced lactate production in macrophages (Figure 6B,C). Glycolysis‐related protein gene expression levels revealed that LPS significantly upregulated these genes, but FE reversed this effect (Figure 6D). Using the Seahorse analyzer to measure the ECAR, we found that LPS increased both basal and compensatory glycolysis levels in macrophages, but FE reversed this trend (Figure 6E–G). Furthermore, analysis of cell culture supernatants demonstrated that FE inhibited LPS‐induced glucose uptake and lactate release by macrophages (Figure 6H,I). These results collectively indicated that FE effectively suppresses LPS‐induced glycolysis activation in macrophages. To further investigate whether FE affects mitochondrial metabolism in macrophages, we measured OCR as an indicator of mitochondrial activity. The results showed that LPS stimulation significantly reduced OCR levels, basal respiration, and maximal respiration in macrophages. However, FE treatment restored maximal respiration but had no significant effect on basal respiration (Figure 6J–L), indicating that FE not only inhibited macrophage glycolysis but also partially restored macrophage mitochondrial function. To explore the underlying reasons for the changes in mitochondrial respiration, we assessed mitochondrial membrane potential using immunofluorescence. LPS stimulation led to a decrease in mitochondrial membrane potential, while FE treatment partially restored it (Figure 6M). Additionally, using Mito‐Tracker Red CMXRos (a mitochondrial red fluorescent probe), we examined changes in mitochondrial morphology. In the control group, mitochondria exhibited a tubular and interconnected structure. However, LPS stimulation caused mitochondrial network fragmentation, resulting in more isolated mitochondria. FE treatment reversed this phenomenon (Figure 6N). These findings collectively demonstrated that FE suppresses the LPS‐induced Warburg effect in macrophages by promoting PKM2 tetramerization and restores mitochondrial respiratory function, thereby alleviating inflammatory responses.

### 2.7 FE Inhibits STAT3/NLRP3 Signaling Pathway by Inhibiting the Protein Kinase Function of PKM2

**Figure 7:**
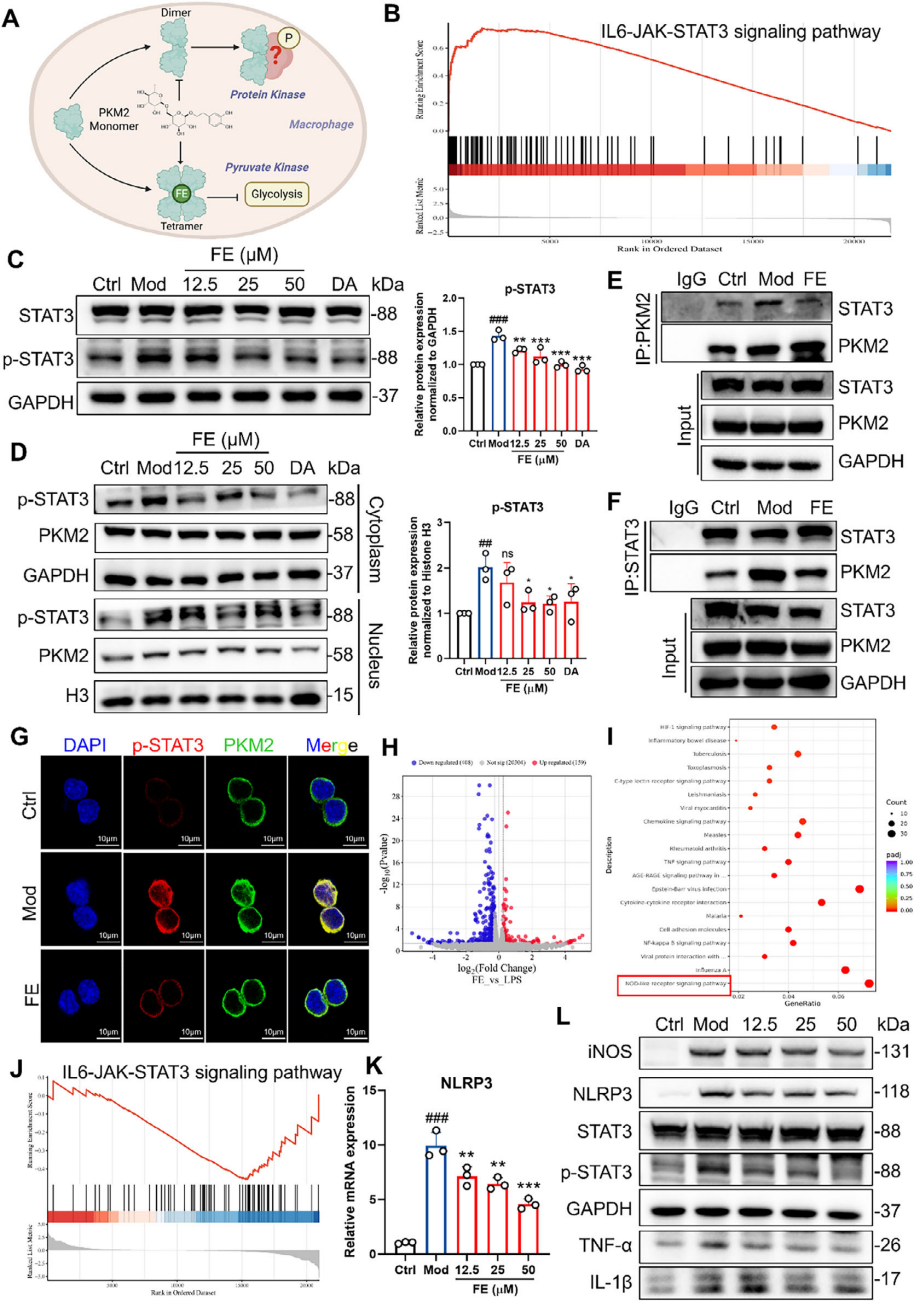
FE Inhibits the STAT3/NLRP3 Signaling Pathway. A) Hypothesis of FE inhibiting PKM2 kinase function. B) GSEA analysis of the IL6‐JAK‐STAT3 pathway in the GSE166488 dataset. C) Expression of P‐STAT3 protein in RAW264.7 cells. D) Western blot analysis of P‐STAT3 expression in cytoplasmic and nuclear fractions. E,F) Co‐IP analysis of interaction between STAT3 and PKM2. G) Immunofluorescence co‐localization of P‐STAT3 and PKM2. H) Volcano plot of differentially expressed genes between FE‐treated and LPS‐induced RAW264.7 cells. I) KEGG pathway enrichment analysis of differentially expressed genes. J) GSEA analysis of the IL‐6‐JAK‐STAT3 pathway between FE‐treated and LPS‐induced RAW264.7 cells. K) mRNA expression of NLRP3 in RAW264.7 cells. L) Western blot analysis of inflammation‐related proteins, P‐STAT3, and NLRP3 in RAW264.7 cells. Data are expressed as the mean ± SD. ^#^ *p* < 0.05, ^##^ *p* < 0.01, ^###^ *p* < 0.001 versus the control group; ^*^ *p* < 0.05, ^**^ *p* < 0.01, ^***^ *p* < 0.001 versus the model group.

In our previous studies, we found that PKM2 predominantly exists in its dimeric form under LPS stimulation. In this state, PKM2 could function as a transcriptional coactivator or protein kinase to mediate the expression of downstream inflammatory factors. Therefore, we aimed to identify the interacting proteins downstream of dimeric PKM2 under LPS stimulation (**Figure** **7**A). First, we performed GSEA on DEGs from the GSE166488 dataset. The results revealed that the IL6‐JAK‐STAT3 signaling pathway was significantly activated in the model group compared to the control group (Figure 7B). Concurrently, cellular experiments showed that the expression of phosphorylated STAT3 (p‐STAT3) was markedly increased under LPS stimulation but significantly decreased after treatment with FE or DASA‐58 (Figure 7C). Further nuclear‐cytoplasmic fractionation assays demonstrated that LPS treatment led to a significant increase in nuclear p‐STAT3 distribution, while FE or DASA‐58 treatment markedly reduced its nuclear expression, a trend consistent with that of PKM2 (Figure 7D). Based on these findings, we hypothesized that the dimeric form of PKM2 interacts with STAT3, leading to its phosphorylation. Co‐IP experiments revealed that, compared to the control group, LPS stimulation significantly increased the interaction between PKM2 and STAT3, while FE effectively inhibited this interaction (Figure 7E,F). Subsequent immunofluorescence assay showed that LPS induction markedly enhanced the fluorescence intensity of p‐STAT3 and promoted its nuclear colocalization with PKM2. However, FE treatment reduced both the fluorescence intensity of p‐STAT3 and its nuclear colocalization with PKM2 (Figure 7G). These results indicated that FE reduces STAT3 phosphorylation by inhibiting the interaction between PKM2 and STAT3. p‐STAT3 has a transcriptional function. To further identify the downstream target proteins affected by FE, we performed transcriptome sequencing to compare differentially expressed genes between the FE treatment group and the LPS stimulation group. KEGG pathway enrichment analysis of these differentially expressed genes revealed that the NOD‐like receptor signaling pathway was significantly enriched (Figure 7H,I). Meanwhile, GSEA analysis of the transcriptome revealed that the IL6‐JAK‐STAT3 signaling pathway was suppressed in the FE‐treated group compared to the LPS‐treated group (Figure 7J). Literature review indicated that STAT3 could regulate the transcriptional level of NLRP3. Subsequent cellular experiments demonstrated that the gene expression level of NLRP3 was significantly upregulated after LPS stimulation but effectively downregulated after FE treatment (Figure 7K). Western blot results showed that, compared to the control group, LPS induction significantly increased the expression of NLRP3, p‐STAT3, and inflammation‐related proteins such as iNOS, TNF‐α, and IL‐1β, while STAT3 levels remained unchanged. After FE treatment, the expression of these proteins was markedly reduced, but STAT3 expression remained unaffected (Figure 7L). The above results indicated that FE alleviates inflammation by inhibiting the interaction between PKM2 and STAT3, suppressing the phosphorylation of STAT3, and the transcriptional activation of downstream NLRP3.

### 2.8 FE Distributed Intact in Serum and Liver without Inducing Multi‐Organ Toxicity

**Figure 8:**
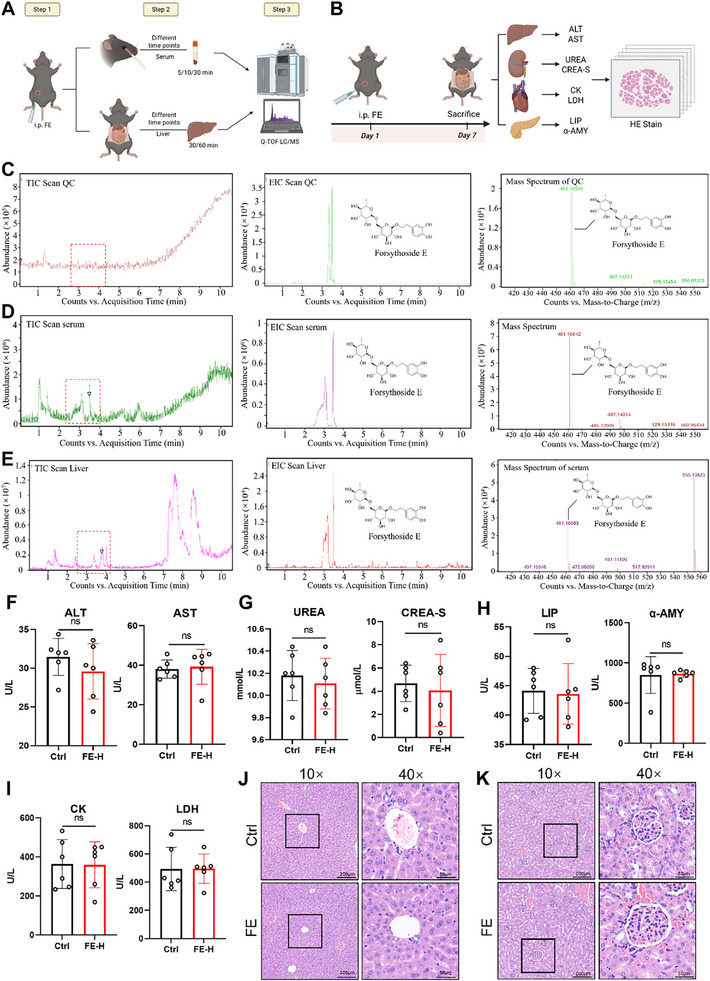
Distribution of FE in Liver and Blood with No Observed Organ Toxicity. A,B) Experimental Design Overview. C) Results of mass spectrometry of FE standards, D) serum samples, and E) liver tissue samples. Detection results of F) liver injury indicators, G) renal injury indicators, H) pancreatic injury indicators, and I)cardiac injury indicators. J) HE staining of the liver and K) kidney.

Since FE has a diglycoside structure, it may react with some metabolic enzyme in vivo, leading to chemical structure changes. So prior to investigating the hepatoprotective effects of FE in mice, this study first examined its distribution in serum and liver tissues, as well as its potential multi‐organ toxicity (**Figure** **8**A,B). By integrating Total Ion Chromatogram (TIC) and Extracted Ion Chromatogram (EIC), FE standard was identified with a retention time of 3–4 min, and its corresponding peak in ESI‐MS matched FE's molecular weight (m/z) (Figure 8C). Analysis of pooled serum and liver samples at different time points revealed a peak at 3–4 min with the same m/z as FE, confirming its prototype distribution in blood and liver (Figure 8D,E). Toxicity was assessed using organ injury biomarkers (liver, kidney, pancreas, heart, etc.). No significant differences were observed between FE‐treated and control groups, indicating no detectable toxicity even at high doses (Figure 8F–I). At the same time, HE staining results showed that there was no significant tissue damage in the liver and kidney in the FE‐treated group (Figure 8J,K). These results have shown that FE distributes intact in murine serum and liver without causing toxicity to other organs, supporting its safety profile for further hepatoprotective studies.

### 2.9 FE Effectively Alleviates Liver Injury in Septic Mice

**Figure 9:**
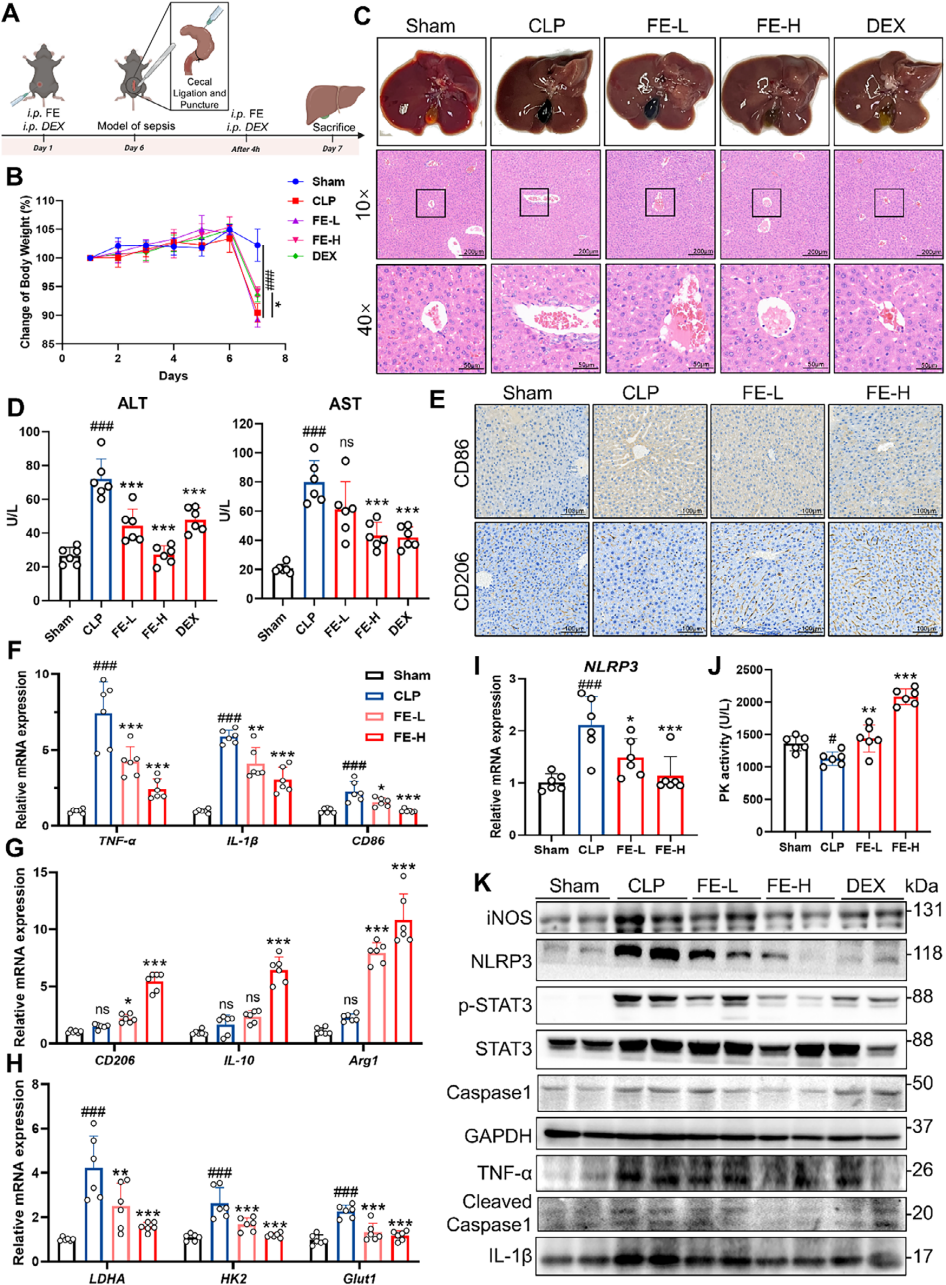
FE Alleviates Sepsis‐Induced Liver Injury in Mice. A) Flow chart of animal experiments. B) Statistical results of weight change. C) Appearance and HE staining of liver tissues from mice. D) Expression of ALT and AST in mouse serum. E) IHC analysis of CD86 (M1 marker) and CD206 (M2 marker) in mouse liver tissues. F) mRNA expression of M1 macrophage markers (TNF‐α, IL‐1β, CD86) in mouse liver tissues. G) mRNA expression of M2 macrophage markers (CD206, IL‐10, Arg1) in mouse liver tissues. H) mRNA expression of glycolysis‐related proteins (LDHA, Glut1, HK2) in mouse liver tissues. I) mRNA expression of NLRP3 in mouse liver tissues. J) Detection of pyruvate kinase activity in liver. K) Western blot analysis of inflammation‐related proteins, p‐STAT3, and NLRP3 in mouse liver tissues. Data are expressed as the mean ± SD. ^#^ *p* < 0.05, ^##^ *p* < 0.01, ^###^ *p* < 0.001 versus the control group; ^*^ *p* < 0.05, ^**^ *p* < 0.01, ^***^ *p* < 0.001 versus the model group.

To evaluate the therapeutic effects of FE on sepsis‐induced liver injury in vivo, we established a sepsis model in mice through CLP (**Figure** **9**A). Compared to the Sham group, both the CLP and treatment groups exhibited significant weight loss in mice. However, the FE‐H group and the DEX group showed attenuated weight reduction relative to the CLP group (Figure 9B). Observations of liver tissue revealed that compared with Sham group, the CLP group displayed dark brown livers with black gallbladders, as well as congested liver edges and overall signs of severe liver injury. Following treatment with FE or DEX, the liver color shifted back to reddish‐brown, the gallbladder turned light green, and liver edge congestion was alleviated, suggesting that FE or DEX mitigated liver injury in septic mice. Further histological analysis of liver tissues using HE staining showed that hepatocytes in the Sham group were arranged radially, with large, round nuclei centrally located and uniform cytoplasm. In the CLP group, hepatocyte arrangement was disordered, with cell swelling, atrophy, nuclear pyknosis, and cytoplasmic vacuolization. These pathological changes were partially reversed after treatment with FE or DEX (Figure 9C). Serum analysis of the mice revealed that, compared to the Sham group, the CLP group exhibited significantly elevated levels of ALT and AST. However, treatment with FE or DEX effectively reduced the expression of ALT and AST in the serum. These results indicate that FE alleviates liver injury in septic mice (Figure 9D). The results of immunohistochemistry showed that compared to the Sham group, the CLP group exhibited a significant increase in CD86 expression, while CD206 levels remained unchanged. However, after treatment with different doses of FE, CD86 expression was suppressed, and CD206 expression gradually increased. This suggests that FE inhibits the polarization of macrophages toward the pro‐inflammatory M1 phenotype while promoting their transformation into the anti‐inflammatory and reparative M2 phenotype (Figure 9E). In vivo data assessed by qPCR analysis revealed that FE significantly decreased the expression of M1 macrophage polarization marker genes, including *Tnf‐α*, *IL‐1β*, and *Cd86*, while increasing the expression of M2 polarization marker genes, such as *Cd206*, *IL‐10*, and *Arg1*. Additionally, the expression of *Nlrp3* and glycolysis‐related genes, including *Ldha*, *Glut1*, and *Hk2*, was also inhibited by FE, aligning with the results from cell experiments (Figure 9F–I). Meanwhile, FE significantly reversed the CLP‐induced decrease in pyruvate kinase activity in mouse liver (Figure 9J). Western blot results showed that, compared to the Sham group, the CLP group exhibited significantly increased expression of NLRP3, p‐STAT3, and inflammation‐related marker proteins such as iNOS, IL‐1β, and TNF‐α. After treatment with FE, the expression of NLRP3, p‐STAT3, and inflammation‐related marker proteins were markedly reduced, consistent with the trends observed in cell experiments (Figure 9K). These findings demonstrate that FE alleviates liver injury in septic mice by regulating macrophage polarization phenotypes and suppressing the STAT3/NLRP3 signaling pathway.

### 2.10 FE Targeting PKM2 K311 in Macrophages Alleviates Sepsis‐Induced Liver Injury

**Figure 10:**
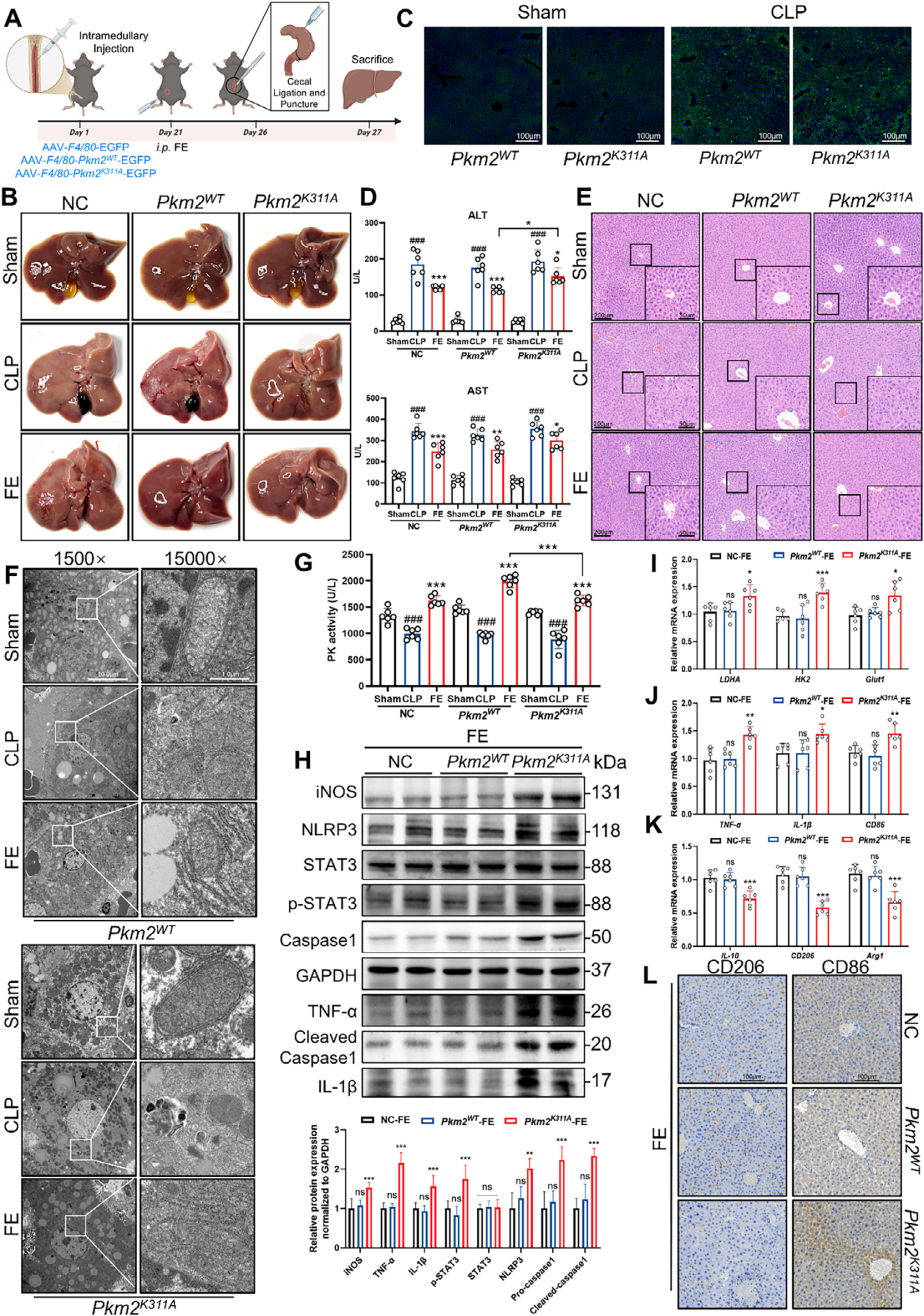
FE Targeting PKM2 K311 in Macrophages Alleviates Sepsis‐induced Liver Injury. A) Flow chart of animal experiments. B) Appearance of mouse liver tissue C) Liver tissue immunofluorescence. D) Expression of ALT and AST in mouse serum. E) HE staining of liver tissues from mice F) Results of transmission electron microscopy of mouse liver tissue. G) Detection of pyruvate kinase activity in liver. H) Western blot analysis of inflammation‐related proteins, p‐STAT3, NLRP3, Pro‐caspase 1, and Cleaved‐caspase 1 in mouse liver tissues. I) mRNA expression of glycolysis‐related proteins (LDHA, Glut1, HK2) in mouse liver tissues. J) mRNA expression of M1 macrophage markers (TNF‐α, IL‐1β, CD86) in mouse liver tissues. K) mRNA expression of M2 macrophage markers (CD206, IL‐10, Arg1) in mouse liver tissues. L) IHC analysis of CD86 (M1 marker) and CD206 (M2 marker) in mouse liver tissues. Data are expressed as the mean ± SD. ^###^ *p* < 0.001 versus the Sham group; ^*^ *p* < 0.05, ^**^ *p* < 0.01, ^***^ *p* < 0.001 versus the CLP group or *Pkm2^WT^* group.

To further validate the mechanism by which FE alleviates sepsis‐induced liver injury through targeting the PKM2 K311 site in macrophages, this study constructed mouse models with macrophage‐specific overexpression of PKM2 WT, PKM2 K311A, and negative control (NC) by intra‐bone marrow injection of adeno‐associated virus 9 (AAV9) carrying *Pkm2^WT^* or *Pkm2^K311A^* genes (**Figure** **10**A). After FE treatment, partial restoration of hepatic ischemia was observed in NC and *Pkm2^WT^* groups, but no significant improvement occurred in the *Pkm2^K311A^* group (Figure 10B). Enhanced EGFP fluorescence in liver tissues following CLP modeling revealed extensive recruitment of bone marrow‐derived macrophages, particularly around central veins, hepatic sinusoids, and inflammatory lesions (Figure 10C). Serum analysis demonstrated reduced ALT and AST levels across all groups post‐FE treatment, with more pronounced ALT reduction in *Pkm2^WT^*‐FE compared to *Pkm2^K311A^*‐FE (Figure 10D). Histopathological evaluation via HE staining showed superior hepatoprotective effects of FE in *Pkm2^WT^*‐FE versus *Pkm2^K311A^*‐FE groups (Figure 10E). Transmission electron microscopy (TEM) revealed that CLP‐induced mice exhibited lipid droplet accumulation, mitochondrial swelling, cristae disruption, and matrix rarefaction. FE treatment restored mitochondrial morphology and reduced lipid droplets in *Pkm2^WT^* group, but not in *Pkm2^K311A^* group (Figure 10F). These findings indicate that macrophage‐specific PKM2 K311A overexpression attenuates FE's therapeutic efficacy. Further mechanistic investigation showed significantly higher hepatic pyruvate kinase activity in *Pkm2^WT^*‐FE than *Pkm2^K311A^*‐FE (Figure 10G). Western blot analysis demonstrated FE's inability to suppress inflammatory markers (iNOS, TNF‐α, IL1‐β), p‐STAT3, NLRP3, and Caspase‐1 activation in *Pkm2^K311A^* group. Consistent with disrupted FE‐PKM2 binding, PKM2 K311A abolished FE's suppression of inflammatory markers. (Figure 10H). Similarly, FE failed to downregulate glycolysis‐related protein mRNA levels in *Pkm2^K311A^* macrophages (Figure 10I). Polarization assays revealed that PKM2 K311A mutation abrogated FE's inhibition of M1 polarization and promotion of M2 polarization in macrophages at both mRNA and protein levels (Figure 10J–L). Collectively, these results demonstrate that FE mitigates sepsis‐associated liver injury by targeting the K311 site of PKM2 in macrophages to promote M2 polarization.

## 3 Discussion

This study reveals for the first time the mechanism by which FE regulates macrophage metabolism and inhibits NLRP3 transcription to alleviate sepsis‐induced liver injury by targeted promotion of PKM2 tetramerization. Based on GEO database analysis and structural virtual screening, we confirmed that FE, as a novel PKM2 tetramerization agonist, reshapes its oligomeric state by binding to the K311 site of PKM2, thereby inhibiting macrophage glycolysis and restoring mitochondrial oxidative phosphorylation function. Furthermore, FE suppresses STAT3 phosphorylation and its downstream NLRP3 transcriptional activation by blocking the PKM2/STAT3 interaction, ultimately driving macrophages toward the M2 anti‐inflammatory phenotype. This discovery not only expands the understanding of PKM2's role in immunometabolic regulation during sepsis but also provides new insights for developing sepsis treatment strategies targeting metabolic reprogramming.

Previous studies have found that the PKM2 dimer maintains the M1 phenotype by enhancing glycolytic flux and nuclear translocation capability, while the tetramer promotes M2 polarization through metabolic reprogramming.^[^ ^16^, ^25^ ^]^ Consistent with this, our study confirmed through targeted pull‐down (Biotin/PLA probes), SPR, and CETSA experiments that FE directly binds to PKM2 and promotes its tetramerization, thereby inhibiting the expression of key glycolytic enzymes (LDHA, HK2, Glut1) and reducing ECAR levels. Notably, FE also enhances OCR levels by restoring mitochondrial membrane potential and morphological remodeling, leading to a shift in macrophage metabolic mode from the Warburg effect to oxidative phosphorylation. This finding is highly consistent with the PKM2 tetramer‐dependent mechanism of metabolic homeostasis regulation.^[^ ^26^ ^]^

In addition to metabolic regulation, this study further reveals the epigenetic regulatory function of FE. Research indicates that NLRP3 activation in macrophages promotes M1 macrophage polarization, thereby exacerbating liver injury.^[^ ^27^ ^]^ This study found that FE treatment significantly reduces the nuclear localization of STAT3 and its co‐localization with PKM2, and transcriptome sequencing confirmed the inhibition of NLRP3 transcription levels. We speculate that FE may inhibit the phosphorylation of STAT3 by PKM2, thereby blocking the activation of NLRP3 promoter by STAT3, which is consistent with a recent study.^[^ ^28^ ^]^ This ‘metabolic remodeling‐epigenetic modification’ synergistic mechanism provides a theoretical basis for explaining the dual anti‐inflammatory effects of FE.

Although significant progress has been made in this study, the following limitations still need to be addressed: first, the glycosyl portion of FE may undergo hydrolysis in vivo, affecting its bioavailability and targeted delivery efficiency. Despite detecting intact FE in vivo, potential hydrolysis warrants further pharmacokinetic studies. Second, the specific regulatory mechanism of PKM2 tetramer on mitochondrial dynamics, such as fusion/fission equilibrium, has not been fully elucidated. In addition, the impact of FE on NLRP3 mediated cell pyroptosis needs further investigation. Future research directions include: I) Design FE prodrug or nano delivery system to enhance its membrane permeability; II) Construct macrophage‐specific PKM2 mutant models and analyze the tetramerization‐dependent metabolic immune regulatory network; III) Explore the combination therapy strategy of FE with other anti‐inflammatory drugs (such as IL‐1β antagonists).

In summary, this study systematically elucidates the mechanism by which FE alleviates sepsis‐induced liver injury through dual mechanisms of PKM2 tetramerization‐mediated metabolic reprogramming and epigenetic regulation. This not only provides experimental evidence for the clinical application of FE but also opens new avenues for sepsis treatment strategies targeting immunometabolic regulation.

## 4 Experimental Section

### Animal Experiments

Male C57BL/6 mice aged 6–8 weeks were obtained from Guangdong Medical Laboratory Animal Center (Guangzhou, China). Macrophage‐specific overexpression of PKM2 WT or PKM2 K311A was achieved through intra‐bone marrow injection of AAV‐*F4/80*‐*Pkm2^WT^/Pkm2^K311A^* adenovirus (1 × 10^13^ vg/kg) for 20 days. All animals were housed in a specific pathogen‐free environment, and experimental protocols were approved by the Animal Care Committee of the International Institute for Translational Chinese Medicine, Guangzhou University of Chinese Medicine (ethical approval number: 20240906).

Mice received daily intraperitoneal injections of FE (Shanghai yuanye Bio‐Technology Co., B20729) at low (20 mg/kg/d) and high doses (80 mg/kg/d), or dexamethasone (DEX, 10 mg/kg/d),^[^ ^24^, ^29^, ^30^ ^]^ while control groups received equivalent volumes of saline based on body weight. After 5 consecutive days of administration, a sepsis model was induced via cecal ligation and puncture (CLP) on day 6. FE or DEX (MCE, HY‐14648) was administered intraperitoneally 4 h post‐CLP, followed by overnight fasting.

### Cell Culture

RAW264.7 macrophages (Shanghai Cell Bank, China) were cultured in DMEM (Gibco) supplemented with 10% fetal bovine serum (FBS, Gibco) and 1% penicillin/streptomycin (Solarbio) at 37 °C under 5% CO_2_. In vitro experiments, cells were pretreated with FE (12.5, 25, 50 µm) or DASA‐58(25 µm, a PKM2 tetramerization agonist, as positive control) for 1 h, followed by LPS (200 ng mL^−1^) stimulation for 12 h.

### Transcriptomic Analysis and Differential Gene Expression

Microarray datasets (GSE57065, GSE166488, GSE254497) were retrieved from the GEO database (https://www.ncbi.nlm.nih.gov/geo/). Differential expression analysis was performed using the following pipelines: GSE166488: Processed with the *limma* package in R (v4.3.1).^[^ ^31^ ^]^ GSE254497: Analyzed using *Seurat* (v4.0) for single‐cell RNA sequencing.^[^ ^32^ ^]^ Differentially expressed genes (DEGs) were identified with thresholds of |log_2_FC| > 1 and *P* < 0.05. Volcano plots and heatmaps were generated using *ggplot2* (v3.4.0) and *pheatmap* (v1.0.12).

### Functional Enrichment Analysis

KEGG pathway and Gene Set Enrichment Analysis (GSEA) were performed using the *clusterProfiler* R package (v4.0).^[^ ^33^ ^]^ Enrichment significance was set at *P* < 0.05.

### Virtual Screening and Molecular Docking

The PKM2 crystal structure (PDB ID: 6B6U) was prepared using Schrödinger Suite.^[^ ^34^ ^]^ The Natural Product Monomer Compound Library (comprising 16 708 compounds), the Traditional Chinese Medicine Monomer Compound Library (comprising 2939 compounds), and the TargetMol Compound Library (comprising 17 681 compounds) were processed using LigPrep to generate their 3D conformations.^[^ ^35^, ^36^ ^]^ Docking simulations were conducted in three stages: high‐throughput virtual screening (HTVS), standard precision (SP), and extra precision (XP).^[^ ^37^, ^38^, ^39^ ^]^ Compounds with XP docking scores <−10 kcal/mol were selected for protein‐ligand interaction fingerprint (PLIF) and structural diversity analyses.

### DSS Crosslinking and Western Blotting

Cells were lysed in RIPA buffer (Beyotime) containing protease inhibitors. For tetramer detection, lysates were treated with 1 µm disuccinimidyl suberate (DSS, Thermo Fisher) on ice for 1 h. Proteins were separated via 10% SDS‐PAGE, transferred to PVDF membranes (Millipore), and probed with antibodies against PKM2 (1:1000, Proteintech, 15822‐1‐AP). Blots were visualized using ECL (Thermo Fisher).

### Quantitative PCR (qPCR)

Total RNA was extracted with TRIzol (Invitrogen), reverse‐transcribed into cDNA (AGbio), and analyzed using SYBR Green qPCR Mix (AGbio). GAPDH served as internal control. Primer sequences are listed in Table S1 (Supporting Information).

### Immunofluorescence

Cells were fixed with 4% paraformaldehyde, permeabilized with 0.5% Triton X‐100, and blocked with 10% BSA. Primary antibodies (PKM2, STAT3) were incubated overnight at 4 °C, followed by Alexa Fluor‐conjugated secondary antibodies (Invitrogen). Nuclei were stained with DAPI. Images were captured using a Leica TCS SP8 confocal microscope.

### Biotin/Photoaffinity Probe Pull‐Down

FE‐Biotin or FE‐PLA probes were incubated with RAW264.7 lysates. Streptavidin magnetic beads (Thermo Fisher) were used for pull‐down, followed by silver staining or Western blotting. Interacting proteins were identified via LC‐MS/MS (Oebiotech, Shanghai).

### Surface Plasmon Resonance (SPR)

PKM2 was immobilized on a CM5 chip (Biacore X100, GE Healthcare). FE (0.1–100 µm) was injected at a flow rate of 30 µL min^−1^. Binding kinetics (KD) were calculated using Biacore Evaluation Software.

### Cellular Thermal Shift Assay and Drug Affinity Responsive Target Stability

For cellular thermal shift assay (CETSA), lysates were heated (40–90 °C) post‐FE treatment, and PKM2 stability was assessed by Western blot. For drug affinity responsive target stability (DARTS), FE‐bound proteins were digested with Pronase E and analyzed via SDS‐PAGE.

### Atomic Force Microscopy Force Spectrum Analysis

Streptavidin magnetic beads were fixed on the cantilever, and the cantilever was immersed in FE‐Biontin solution at room temperature to realize the tip functionalization. The purified recombinant protein was absorbed 20 µL and placed on mica sheet for 10 min at room temperature. The cantilever and mica sheet were mounted on an atomic force microscope for force spectrum analysis. The atomic force microscopy (AFM) force spectrum curve was analyzed using JPKSPM data processing software.

### Construction of PKM2 Plasmid

X*ho*I and *Nhe*I restriction enzyme sites were added to the nucleotide primers used for PCR. The primers used were as follows: forward, 5’‐GCGGCAGCGCTAGCATGCCGAAGCCACACAGTGAA‐3'; reverse, 5'‐TTGCACTTCTCGAGTCAAGGTACAGGCACTACACG‐3'. The gene fragment encoding mouse PKM2 (AA1‐531) was amplified from mouse cDNA. The target fragment and the pET28a (+) vector were digested with *Xho*I and *Nhe*I, and the target gene was ligated into the vector at a ratio of 3:1 (target fragment to vector). The plasmid was evaluated by colony PCR and sequencing.

### Protein Expression and Purification

The constructed plasmid was transformed into Escherichia coli BL21(DE3) cells for expression. The cells were inoculated into LB medium and cultured at 37 °C until the optical density at 600 nm (OD_600_) reached 0.6–0.8. Protein expression was induced with 0.2 mm isopropyl β‐D‐1‐thiogalactopyranoside at 25 °C for 10 h. The cells were resuspended in pre‐chilled lysis buffer (40 mm Tris‐HCl pH 8.0, 250 mm NaCl, 100 mm KCl, 10 mm MgCl_2_, 10 mm imidazole, 5 mm β‐mercaptoethanol, and 1% PMSF) and homogenized using a homogenizer at 4 °C and 15 kPSi for three cycles. The PKM2 protein was purified using Ni‐NTA affinity resin and eluted with pre‐chilled elution buffer (40 mm Tris‐HCl pH 8.0, 250 mm NaCl, 100 mm KCl, 10 mm MgCl_2_, 250 mm imidazole, and 1% PMSF). The eluted fractions containing the target protein were concentrated at 4 °C using an Amicon Ultra‐15 centrifugal filter device and loaded onto a Superdex 200 26/60 size‐exclusion column pre‐equilibrated with SEC buffer (20 mm Tris‐HCl pH 8.0, 150 mm NaCl, 100 mm KCl, 10 mm MgCl_2_). The target protein was eluted with SEC buffer.

### Dynamic Light Scattering (DLS)

The purified PKM2 monomeric recombinant protein was obtained using a Superdex 200 26/60 size‐exclusion column. The protein was diluted with SEC buffer to a final concentration of 5 mg mL^−1^. FE was added, and the mixture was incubated on ice for 1 h. The sample was loaded into a Uni‐well (UNchained Labs, Malvern, UK) and analyzed using dynamic light scattering (DLS) on the UNcle system (UNchained Labs, Malvern, UK). Data was processed using the UncleAnalysis software v5.03 (Unchained Labs).

### Fluorescence Resonance Energy Transfer (FRET)

The protein peak corresponding to the PKM2 monomer was collected using a Superdex 200 Increase 10/300 GL gel filtration chromatography column. The recombinant proteins were prepared into a protein solution with a molar concentration of 2 µm per 50 µL. The samples were divided into two groups: one group was supplemented with FAM‐NTA‐NI dye, while the other group received RB‐NTA‐NI dye, both at a final concentration of 500 µm. The mixtures were incubated on ice for 1 h to allow dye binding to the His‐tag. Unbound dyes were removed by ultrafiltration using a centrifugal filter (4 °C, 4,000 rpm, 10 min). The two groups were then mixed at a 1:1 volume ratio, followed by the addition of Forsythoside E and further incubation on ice for 1 h. Subsequently, 100 µL of the mixed solution was transferred to each well of a black 96‐well plate. Fluorescence spectral analysis was performed using a microplate reader with the following parameters: excitation wavelength was 480 nm, and the emission wavelength ranged from 500 to 700 nm.

### Analysis of Lactate Production in Single Cells

RAW264.7 cells were seeded at a density of 10^4^ cells per well in confocal dishes and cultured overnight. After washing the cells three times with PBS, the cell culture medium was replaced with serum‐free medium. The L‐Lactate Assay Kit (Abcam, UK) was used for detection. The reagent was loaded into a hollow probe, and the probe tip was inserted into the cells. Fluorescence intensity and photon counts were measured using a real‐time single‐cell multimodal analyzer equipped with a fiber optic probe (Rayme, China).

### Mitochondrial and Glycolytic Function Assays

Oxygen consumption rate (OCR)/extracellular acidification rate (ECAR): Measured using the Seahorse XF96 Analyzer (Agilent) with the XF Cell Mito Stress and Glycolysis Stress Kits.

### Histopathology and Immunohistochemistry (IHC)

Liver tissues were fixed in 4% paraformaldehyde, paraffin‐embedded, and sectioned (4–6 µm). IHC for CD86 (M1) or CD206 (M2) was performed using standard protocols.^[^ ^40^ ^]^

### Hematoxylin‐Eosin (HE) Staining

Before staining, the mouse liver tissue was washed with saline and fixed with 4% paraformaldehyde. After embedding the tissue in paraffin, it was sectioned to a specific thickness (approximately 4–6 µm), and hematoxylin and eosin (H&E) staining was performed on the tissue.^[^ ^41^ ^]^ All images were captured at random fields of view under an optical microscope (NIKON Eclipse, Japan) at 100× and 400× magnification.

### Co‐Immunoprecipitation (Co‐IP)

Cells were lysed in immunoprecipitation lysis buffer on ice for 1 h, followed by centrifugation at 4 °C and 14 000 rpm for 10 min to collect the supernatant. To 500 µL of the protein supernatant, IgG or PKM2 antibody was added, and the mixture was incubated with shaking at 4 °C overnight. Subsequently, 30 µL of 50% agarose protein A+G was added to each group, and the mixture was shaken at 4 °C for 6 h. After centrifugation at 4 °C and 1000 rpm for 5 min, the supernatant was removed, and the precipitate was washed three times with lysis buffer. The precipitate was resuspended in 2× loading buffer and heated in a metal bath at 100 °C for 10 min. The input group without IgG or PKM2 antibody served as the control. The samples were separated using SDS‐PAGE.

### Statistical Analysis

Data are presented as mean ± SD. Statistical significance was determined by Student's *t*‐test or one‐way ANOVA using GraphPad Prism 9.0 (*P*< 0.05).

## Conflict of Interest

The authors declare no conflict of interest.

## Author Contributions

B.W. and X.Y. are co‐first authors and contributed equally to this work. B.W. and X.Y. performed the experiments. C.W., Z.L., and F.Z. designed the projects and revised the papers. B.W., X.Y., and Y.L. were responsible for data statistics and informatics analysis; L.A., R.Z., and R.Z. modified the article format; B.W. wrote the paper. All authors read and approved the final paper.

## Supporting information



Supporting Information

## Data Availability

The data that support the findings of this study are available from the corresponding author upon reasonable request.
