# Supplementary material for: Forsythoside E Alleviates Liver Injury by Targeting PKM2 Tetramerization to Promote Macrophage M2 Polarization
Source: Adv Sci (Weinh). 2025 Nov 14;13(6):e14514. doi: 10.1002/advs.202514514 (PMC12866822; doi:10.1002/advs.202514514)
Supplement: Supplementary file 1 — Supporting Information [file ADVS-13-e14514-s001.docx]

**Supplementary Figures**

**Forsythoside E alleviates liver injury by targeting PKM2 tetramerization to promote macrophage M2 polarization**

**The PDF file includes:**

Supplementary Figure 1 to 6

**Supplementary Figure**

**
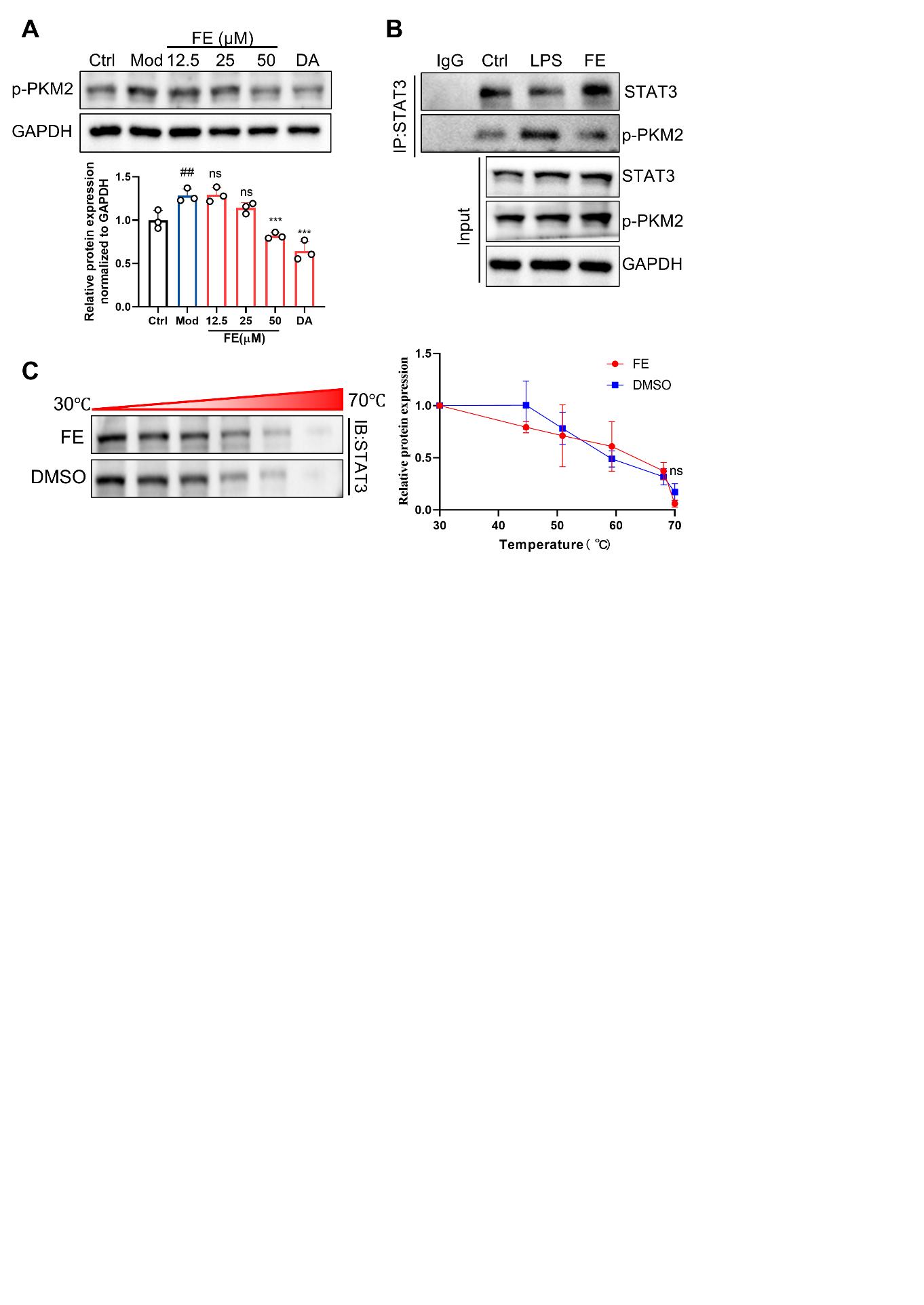
**

**Supplementary Figure 1. FE inhibits the interaction between p-PKM2 and STAT3.** (A) Effect of FE on phosphorylation of PKM2. (B) Co-IP analysis of interaction between STAT3 and p-PKM2. (C) CETSA analysis of thermal stability of STAT3 protein. Data are expressed as the mean ± SD. ^##^*p*<0.01 vs. the control group; ****p*<0.001 vs. the model group.


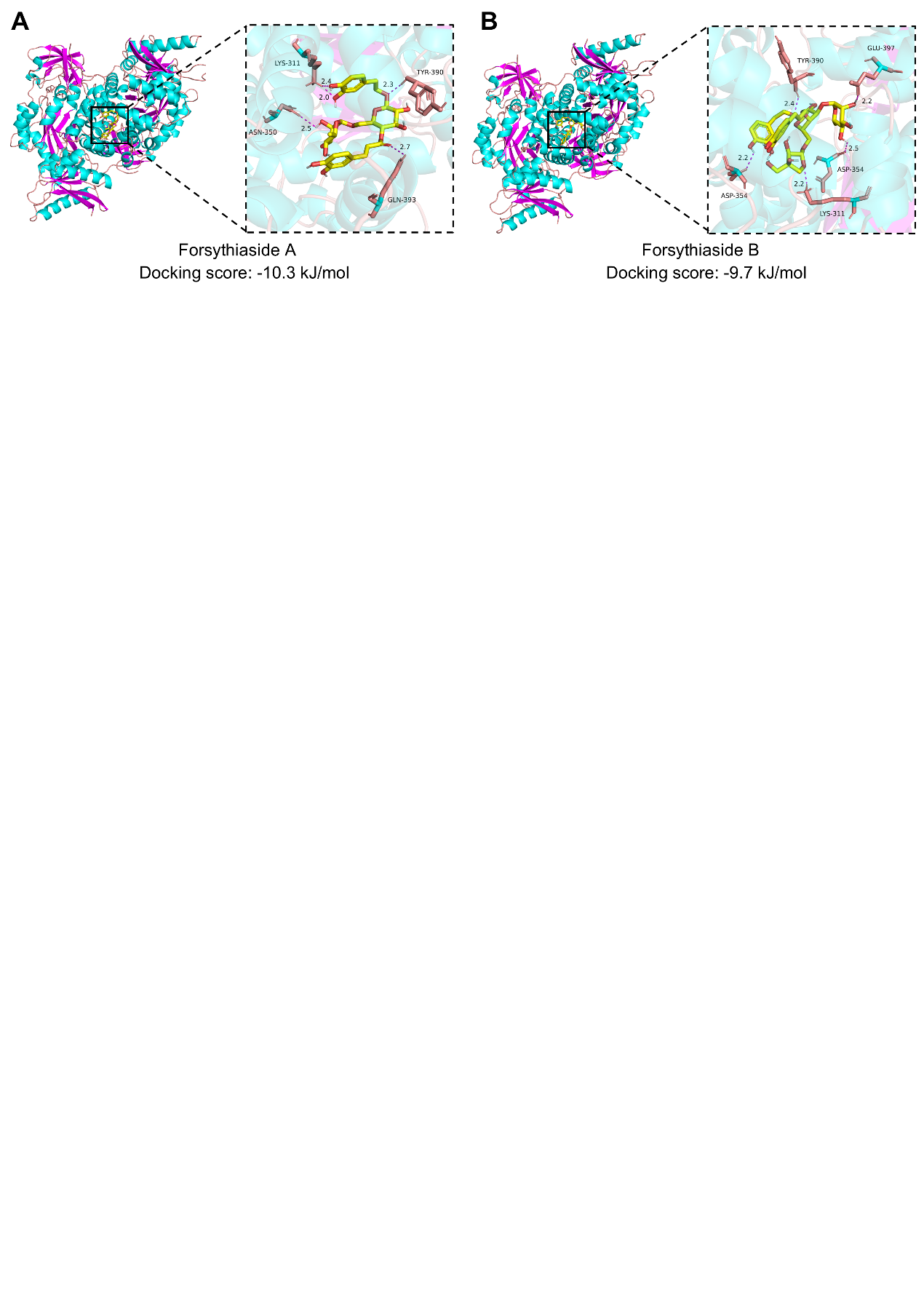


**Supplementary Figure 2. PKM2 has potential interactions with FA and FB.** (A) PKM2-Forsythiaside A binding site map. (B) PKM2- Forsythiaside B binding site map.


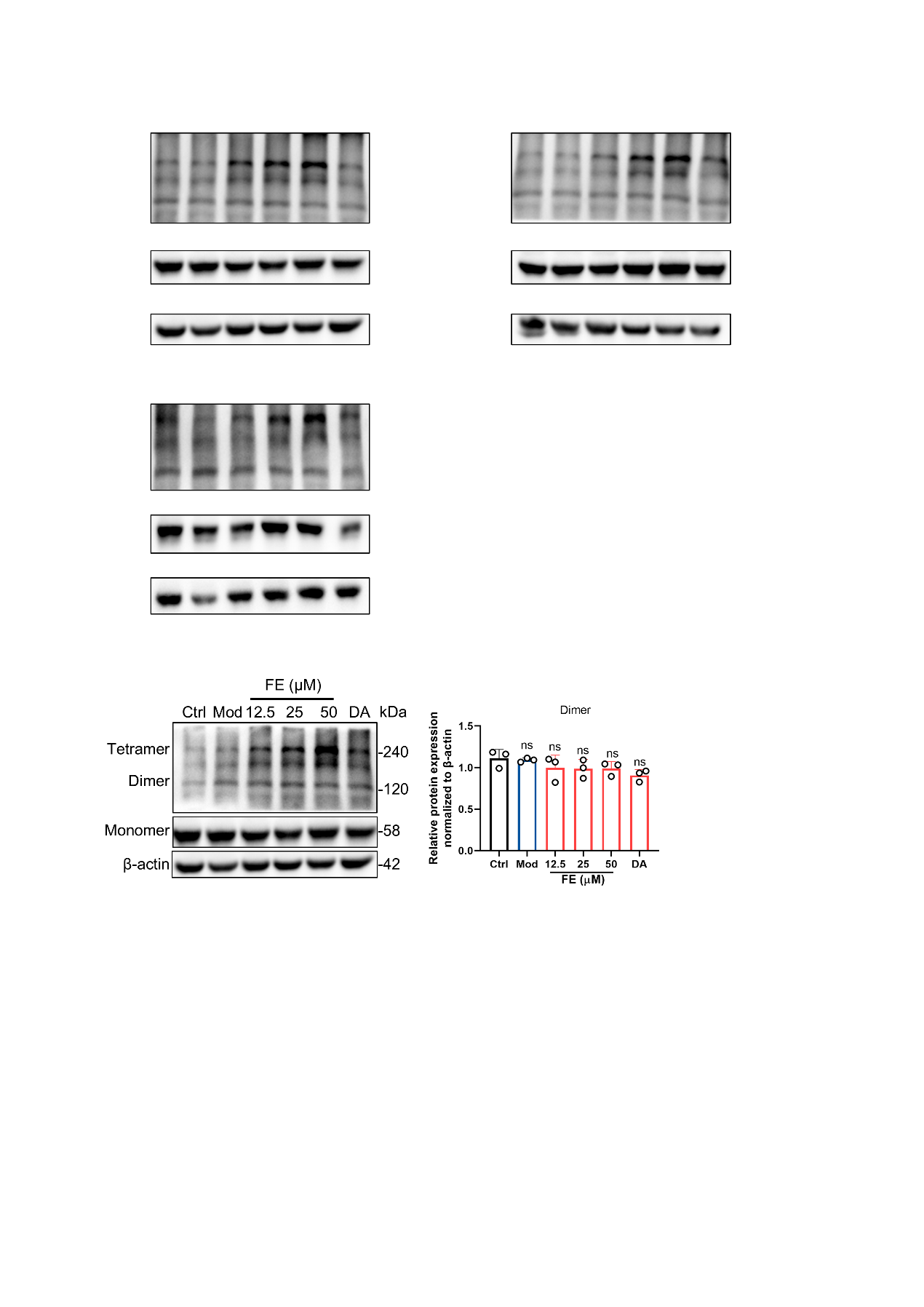


**Supplementary Figure 3. FE promotes PKM2 tetramerization.** Expression of PKM2 dimers in LPS-induced macrophages treated with FE (12.5, 25, 50 μM) or DA (DASA-58, 20 μM).


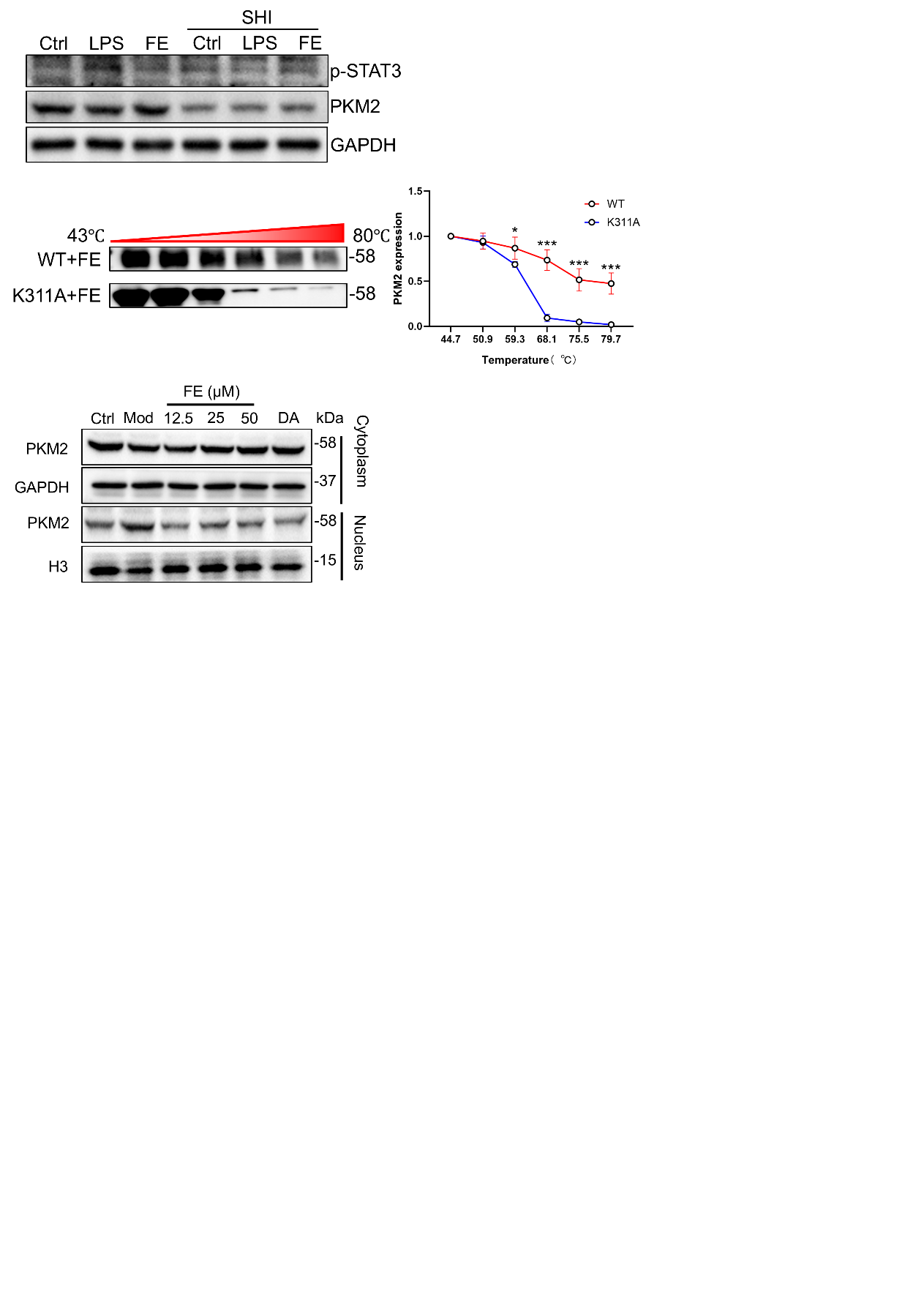


**Supplementary Figure 4.** PKM2 regulates STAT3 phosphorylation.


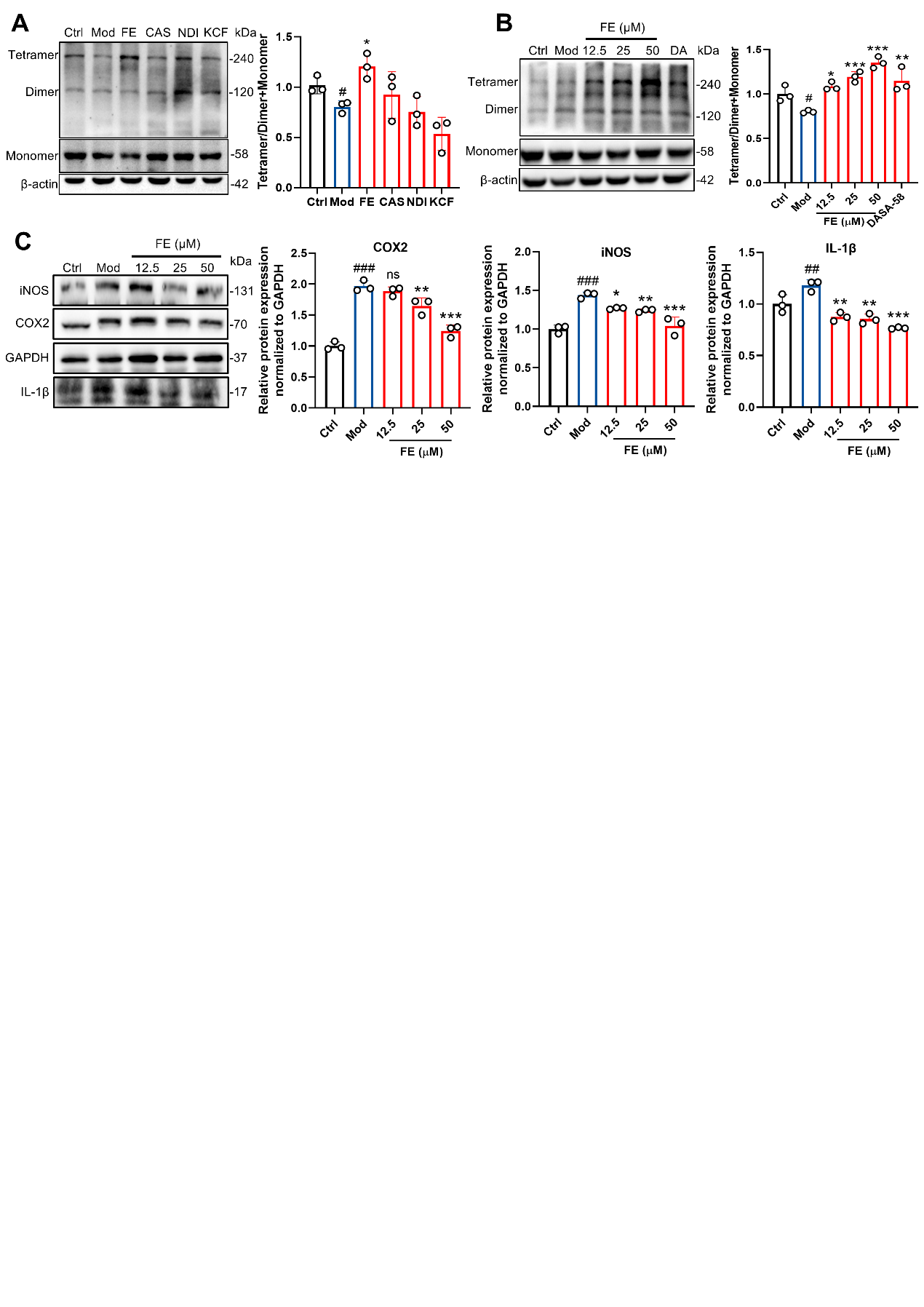


**Supplementary Figure 5. FE is a Potential Allosteric Activator.** (A) DSS crosslinking analysis of PKM2 multimers in RAW264.7 cells treated with four compounds. (B) Expression of PKM2 multimers in LPS-induced macrophages treated with FE (12.5, 25, 50 μM) or DA (DASA-58, 20 μM). (C) Western blot analysis of inflammation-related proteins (iNOS, COX2, IL-1β) in LPS-induced RAW264.7 cells.


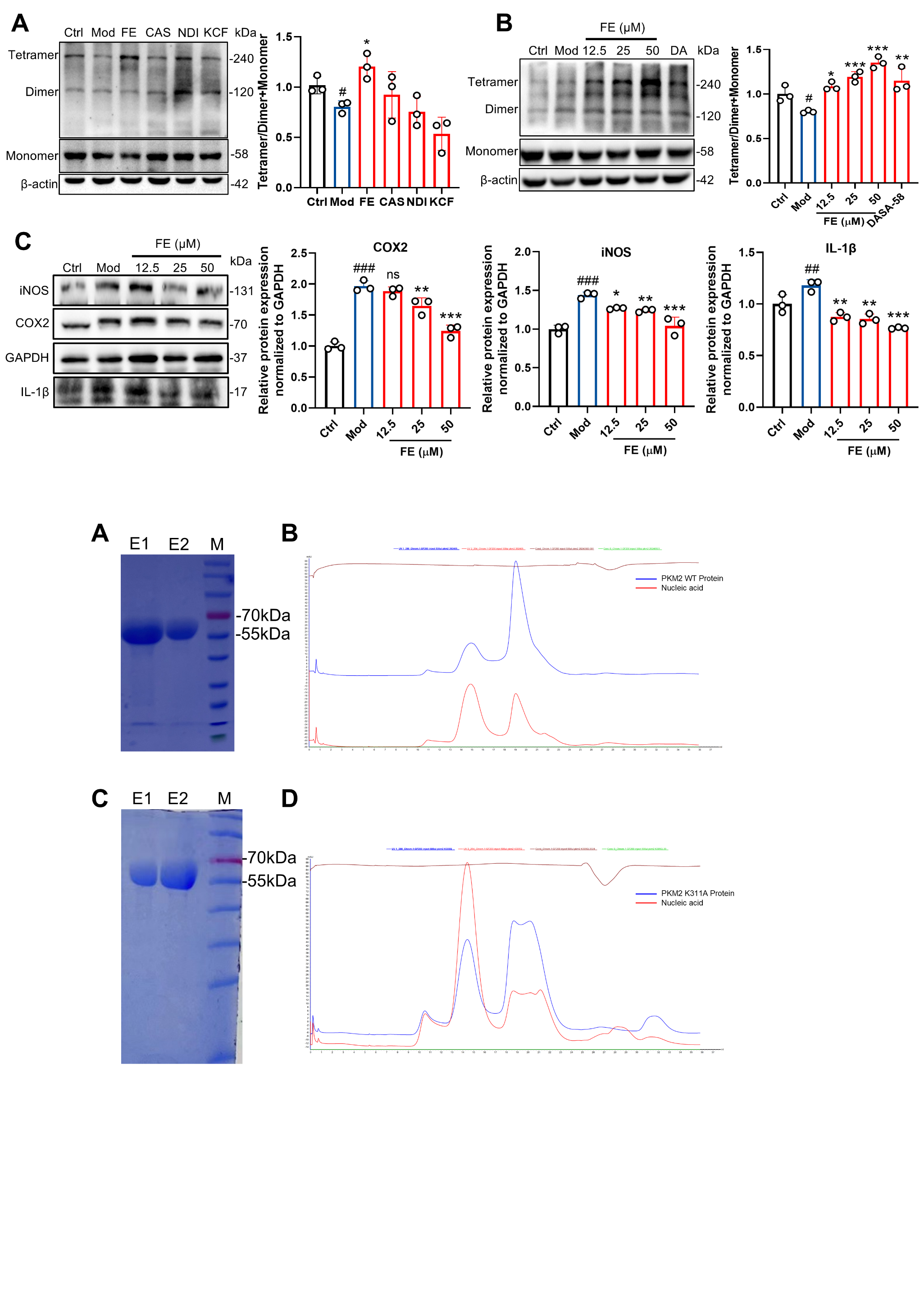


**Supplementary Figure 6. Expression and purification of PKM2 protein.** (A) PKM2 WT protein expression was detected by SDS-PAGE (E1-E2: target protein; M: protein Marker). (B) Chromatogram of PKM2 WT protein after purification on a gel filtration column (). (C) PKM2 K311A protein expression was detected by SDS-PAGE (E1-E2: target protein; M: protein Marker). (D) Chromatogram of PKM2 K311A protein after purification on a gel filtration column.

**Table S1** Real-time PCR primer sequences.

| **Gene** | **Primer sequences.** |
| --- | --- |
| GAPDH | forward, 5′- AAGAAGGTGGTGAAGCAGG-3′,  reverse, 5′- GAAGGTGGAAGAGTGGGAGT-3′. |
| CD206 | forward, 5′- CTCTGTTCAGCTATTGGACGC-3′,  reverse, 5′- CGGAATTTCTGGGATTCAGCTTC-3′. |
| IL-10 | forward, 5′- GCTCTTGCACTACCAAAGCC-3′,  reverse, 5′- CTGCTGATCCTCATGCCAGT-3′. |
| Arg1 | forward, 5′- AACCATCTGGGGCATCACAG-3′,  reverse, 5′- ACCAGAAAGGAACTGCTGGG-3′. |
| CD86 | forward, 5′- ATATGACCGTTGTGTGTGTTCTGGA-3′,  reverse, 5′- AGGGCCACAGTAACTGAAGCTGTAA-3′. |
| IL-1β | forward, 5′- CTTCAGGCAGGCAGTATCACTC-3′,  reverse, 5′- TGCAGTTGTCTAATGGGAACGT-3′. |
| TNF-α | forward, 5′- CAGGCGGTGCCTATGTCTC-3′,  reverse, 5′- CGATCACCCCGAAGTTCAGTAG--3′. |
| iNOS | forward, 5′- CAGGAGGAGAGAGATCCGATTTA-3′,  reverse, 5′- GCATTAGCATGGAAGCAAAGA--3′. |
| NLRP3 | forward, 5′- TCACAACTCGCCCAAGGAGGAA-3′,  reverse, 5′- AAGAGACCACGGCAGAAGCTAG-3′. |
| LDHA | forward, 5′- TGTCTCCAGCAAAGACTACTGT-3′,  reverse, 5′- GACTGTACTTGACAATGTTGGGA-3′. |
| Glut1 | forward, 5′- CAGTTCGGCTATAACACTGGTG-3′,  reverse, 5′- GCCCCCGACAGAGAAGATG-3′. |
| HK2 | forward, 5′- CTTACCGTCTGGCTGACCAACAC-3′,  reverse, 5′- CTCCATTTCCACCTTCATCCTTCT-3′. |
